# Gut–Bone Axis Mediates Exercise Modality‐Dependent Suppression of Inflammatory Osteoclastogenesis in Ovariectomy‐Induced Bone Loss

**DOI:** 10.1155/mi/5715332

**Published:** 2025-12-12

**Authors:** Yucheng Gao, Hao Wang, Liu Shi, Mumin Cao, Xiaoyu Liu, Yuanwei Zhang, Xiangxu Chen, Yijun Rong, Bowen Han, Panpan Lu, Guangchun Dai, Wenbin Fan, Yunfeng Rui, Yingjuan Li

**Affiliations:** ^1^ Department of Orthopaedics, Affiliated ZhongDa Hospital, Medical School of Southeast University, Nanjing, Jiangsu Province, China, seu.edu.cn; ^2^ Orthopaedic Trauma Institute (OTI), Southeast University, Nanjing, Jiangsu Province, China, seu.edu.bd; ^3^ Trauma Center, Affiliated ZhongDa Hospital, Medical School of Southeast University, Nanjing, Jiangsu Province, China, seu.edu.cn; ^4^ Multidisciplinary Team (MDT) for Geriatric Hip Fracture Management, Affiliated ZhongDa Hospital, Medical School of Southeast University, Nanjing, Jiangsu Province, China, seu.edu.cn; ^5^ Medical School of Southeast University, Nanjing, Jiangsu Province, China, seu.edu.cn; ^6^ Department of Orthopaedics, Xinhua Hospital, Medical School of Shanghai JiaoTong University, Shanghai, China; ^7^ Department of Geriatrics, Affiliated ZhongDa Hospital, Medical School of Southeast University, Nanjing, Jiangsu Province, China, seu.edu.cn

**Keywords:** bone loss, exercise, gut microbiota, ovariectomy, postmenopausal osteoporosis

## Abstract

Exercise is crucial for postmenopausal osteoporosis (PMOP) management, yet the comparative efficacy of different exercise modalities and the underlying mechanisms remain unclear. This study investigated the differential effects of distance‐matched high‐intensity interval training (HIIT) and moderate‐intensity continuous exercise (MICE) on ovariectomy (OVX)‐induced osteoporosis (OP) in mice. After 12 weeks of training, micro‐CT analysis revealed that MICE, but not HIIT, significantly attenuated OVX‐induced bone loss and microstructural deterioration. Crucially, only MICE suppressed osteoclastogenesis and reduced proinflammatory factors (interleukin [IL]‐6, IL‐1β, and tumor necrosis factor‐alpha [TNF‐α]) expression in the femur, serum, and colon. Mechanistically, MICE uniquely restored gut microbiota (GM) diversity, mitigated dysbiosis, and enhanced intestinal barrier integrity by upregulating the expression of tight junction proteins (TJPs; ZO‐1, occludin, and claudin‐1), thereby reducing systemic inflammation. In contrast, HIIT failed to ameliorate GM imbalance and intestinal permeability. Our findings demonstrate that the protective effect of MICE on OVX‐induced OP is mediated through the gut–bone axis by modulating GM, repairing the intestinal barrier, and suppressing inflammatory osteoclast activation. This study provides novel evidence that the benefits of exercise on PMOP are modality‐dependent, highlighting MICE as a superior strategy and offering mechanistic insights for optimizing exercise prescriptions.

## 1. Introduction

Osteoporosis (OP) is a systemic skeletal disorder characterized by decreased bone mass, microarchitectural deterioration of bone tissue, and consequent increased bone fragility [1]. Among its various forms, postmenopausal OP (PMOP), the most prevalent subtype of primary OP, represents a chronic metabolic bone disease predominantly caused by estrogen deficiency following menopause. This condition results from the complex interplay of multiple factors, including ethnicity, aging, genetic predisposition, and nutritional status [2]. Epidemiologic studies indicate that PMOP affects upto 32.1% of women over 50 years old, with aging, estrogen deficiency, persistent calcium loss, and smoking identified as independent risk factors [3]. PMOP‐related fragility fractures predominantly occur in the vertebrae, proximal humerus, wrist, and hip, with hip fractures demonstrating the most severe clinical outcomes, including high rates of disability and mortality, along with substantial socioeconomic burdens [4].

The gut microbiota (GM) and its metabolites, constituting the largest and most intricate microecosystem in humans, play pivotal roles in modulating host metabolism, nutrient absorption, and immune regulation [5]. Mounting evidence indicates that dysregulated physiological metabolism is influenced by the maintenance of GM homeostasis [6]. Environmental and physiological perturbations can disrupt this delicate microbial equilibrium, predisposing individuals to various inflammatory and metabolic disorders, including inflammatory bowel disease, colorectal cancer, obesity, diabetes mellitus [7–9], and OP [10]. Estrogen deficiency has been shown to reduce the abundance of immunomodulatory GM species (e.g., *Clostridium* spp., *Lactobacillus acidophilus*, and *Bacillus coagulans*) that suppress proinflammatory cytokine expression, thereby elevating systemic inflammation and accelerating bone loss [11]. These microbial populations exert osteoprotective effects partly by modulating Treg/Th17 cell balance. Th17 cells promote osteoclastogenesis through secretion of key cytokines, including receptor activator of nuclear factor kappa‐B ligand (RANKL) and tumor necrosis factor‐alpha (TNF‐α), whereas Treg cells suppress osteoclast differentiation and maturation via interleukin‐10 (IL‐10) and IL‐4 production [12, 13]. Collectively, these findings corroborate our previous reviews in substantiating the critical involvement of the gut–bone axis in PMOP pathogenesis, positioning GM modulation, and intestinal homeostasis maintenance as promising therapeutic targets for PMOP management [14–16].

Clinically recommended comprehensive management strategies for PMOP encompass exercise interventions, smoking/alcohol cessation, calcium and vitamin D supplementation, and antiosteoporotic pharmacotherapy [17, 18]. Among these, rational exercise regimens serve as a foundational and pivotal strategy for PMOP control [19, 20]. Furthermore, physical activity represents one of the most accessible, reliable, and cost‐effective therapeutic interventions for PMOP prevention and management [15]. Nevertheless, the precise mechanisms underlying exercise‐mediated osteoprotective effects remain incompletely elucidated, and current exercise prescription guidelines for PMOP patients lack robust evidence‐based standardization.

Appropriate exercise modalities modulate GM diversity and enriches beneficial microbial populations within the intestinal ecosystem [21]. This physiological cascade augments gastrointestinal motility, facilitates timely fecal excretion, and consequently reduces colonic mucosal exposure time to pathogens and toxic metabolites. Such mechanical and biochemical modifications ultimately modulate luminal content characteristics and reshape GM composition [22]. Furthermore, accumulating evidence demonstrates that exercise‐induced modifications in intestinal barrier integrity, coupled with structural and compositional remodeling of the GM, may fundamentally regulate disease pathogenesis across multiple systems [23–25].

Previous studies have shown some controversy regarding the potential of exercise to alleviate PMOP and OVX‐induced bone loss [26–29]. This inconsistency may be attributed to variations in exercise protocols used across different studies, and the mechanisms underlying these differences remain unclear. This highlights the fact that not all exercise modalities may be suitable for the prevention and treatment of bone loss caused by estrogen deficiency. The exploration of the most beneficial exercise intensity for orthopedic health seems to be an issue yet to be addressed in clinical practice [30].

In previous review, we proposed a novel hypothesis that gut homeostasis maintenance, mediated by GM and its metabolic derivatives, intricately participates in exercise‐induced osteoprotective regulation through the gut–bone axis [31]. Current evidence reveals a sophisticated bidirectional interaction between exercise regimens and GM dynamics, wherein differential microbial responses are observed across diverse physical activity paradigms [32]. This variability is principally governed by multidimensional intervention parameters, including, but not limited to, exercise typology, intensity stratification, temporal patterns, periodicity, and environmental determinants [33, 34]. Importantly, such exercise‐specific microbial regulation may differentially modulate intestinal barrier competence and GM ecosystem stability, thereby generating heterogeneous impacts on PMOP pathogenesis through distinct microbiome‐gut‐circulation‐bone cascades.

In this study, we utilized the well‐established ovariectomy (OVX)‐induced PMOP mouse model to compare the effects of different exercise interventions‐high‐intensity interval training (HIIT) and moderate‐intensity continuous exercise (MICE)‐against trabecular microarchitecture deterioration and bone mass loss. Our comparative analysis revealed that MICE, rather than HIIT, conferred significant protection against OVX‐induced osteopenia and bone microarchitecture deterioration. This protective effect is likely mediated through the gut–bone axis, by ameliorating GM dysbiosis, restoring gut epithelial integrity, optimizing intestinal mucosal permeability, suppressing systemic inflammation via cytokine downregulation and inhibiting osteoclast activation. These findings collectively elucidate the pathophysiological mechanisms through which exercise modalities differentially regulate PMOP progression, while providing translational evidence for optimizing exercise prescriptions in PMOP prophylaxis.

## 2. Materials and Methods

### 2.1. Ethics, Preparation, and Handling Related to Animals

The C57BL/6 mice (8 weeks old) were purchased from Nanjing Cavans Biotechnology Co., Ltd. (China). They were housed under specific pathogen‐free (SPF) conditions and had unrestricted access to food and water, with the housing environment maintained at 25 ± 2°C, 50% ± 5% humidity, and a 12‐h light/12‐h dark cycle. After a 1‐week acclimatization period, the mice were used for experimentation. The animal experimental design adhered to the guidelines for the care and use of laboratory animals provided by the Southeast University Animal Care and Use Guidelines, and the study protocol was approved by the Institutional Animal Care and Use Committee (IACUC) of the School of Medicine, Southeast University (Approval Number: 20240301034).

### 2.2. Establishment of the OVX‐Induced PMOP Mouse Model

The OVX‐induced PMOP mouse model was established following previously established protocols [35–37]. Mice were fasted for 8 h and then anesthetized with a 1% sodium pentobarbital solution (40 mg/kg). They were placed in a prone position, and the surgical area was shaved. A longitudinal incision was made approximately 1 cm lateral to the midline on both sides of the back. Using tissue scissors, the muscle layers were separated to locate, identify, ligate, and remove both ovaries. Hemostasis was achieved using cotton swabs, and the incision was sutured layer by layer with absorbable sutures. After the procedure, the mice were placed on a warming pad to recover. Within 24 h post‐surgery, the mice were provided with sterile drinking water containing penicillin–streptomycin and celecoxib to prevent infection and alleviate pain.

### 2.3. Animal Experiment Design

The schematic diagram of the animal experiment design is shown in Figure 1. As illustrated in Figure 1A, after 1 week of environmental adaptation, 32 mice were randomly divided into four groups, including the Sham group, OVX group, OVX‐HIIT group, and OVX‐MICE group, with eight mice in each group. For the Sham group, the ovaries were preserved, and an equivalent amount of bilateral ovarian fat pads were excised. Mice in the OVX group underwent bilateral OVX, with all other procedures the same as the Sham group.

Figure 1The schematic representation of experiments and effect of exercise intervention on physiological indices of mice with ovariectomy (OVX)‐induced osteoporosis (OP). (A) Schematic diagram of animal treatment. (B) Body weight changes in each group of mice during the experimental period. (C) Percentage of body weight increase in each group. (D) Percentage of grip strength increase in each group. (E) Estrogen levels prior to exercise intervention in each group of mice. Data represent the mean ± SD, *n* = 8 per group.  ^∗^ *p* < 0.05,  ^∗∗^ *p* < 0.01,  ^∗∗∗^ *p* < 0.001, and  ^∗∗∗∗^ *p* < 0.0001 compared with the corresponding group. HIIT, high‐intensity interval training; MICE, moderate‐intensity continuous exercise; ns, no significance.

**Figure:**
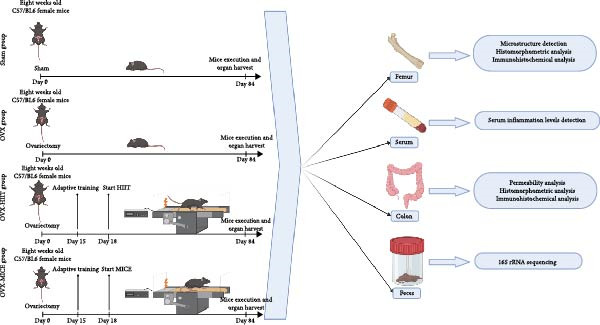
(A)

**Figure:**
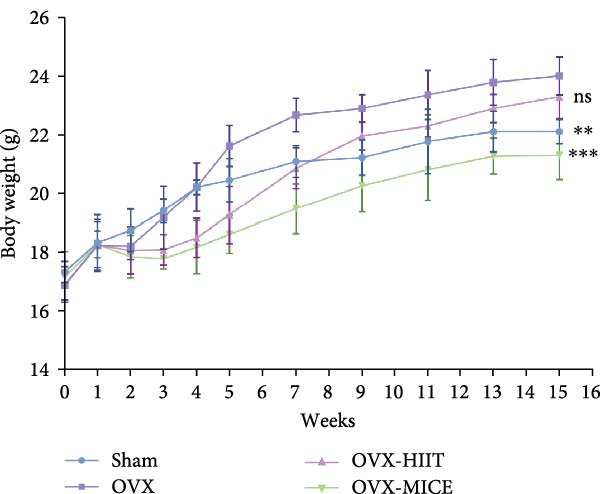
(B)

**Figure:**
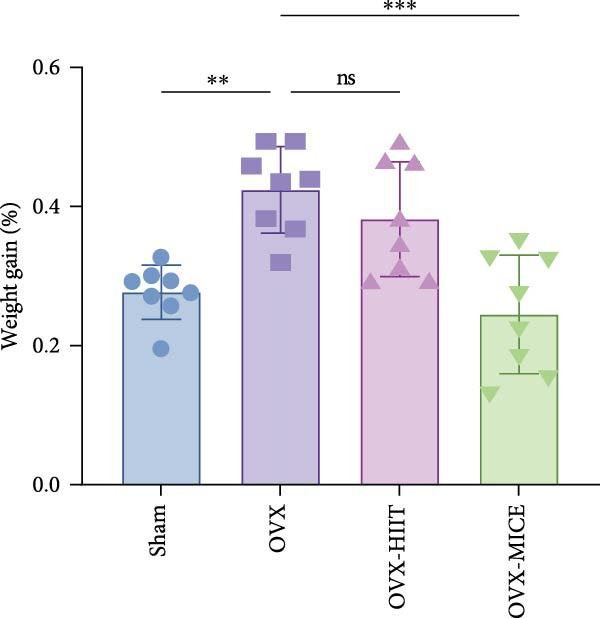
(C)

**Figure:**
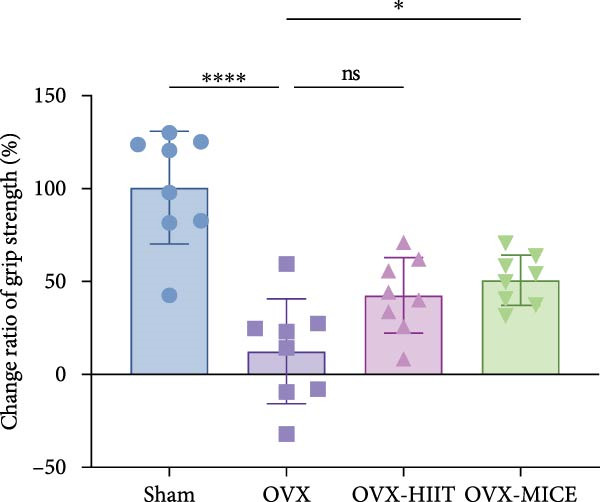
(D)

**Figure:**
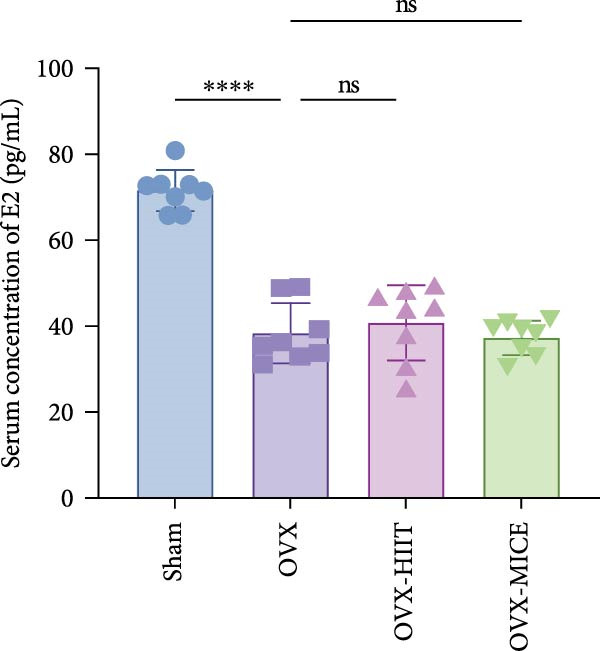
(E)

Based on the OVX model, the OVX‐HIIT and OVX‐MICE groups began treadmill‐based adaptive training starting at week 4 post‐OVX, and from week 5 until the day before euthanasia, these groups continued with treadmill interventions for 5 days per week for a total of 12 weeks.

At the end of the experimental period, the mice were fasted for 12 h, and fecal samples were collected and stored at −80°C. The following day, the mice were euthanized. The euthanasia procedure was performed as follows: mice were first anesthetized with isoflurane (5% concentration in oxygen at 1 L/min flow rate) in an induction chamber until loss of righting reflex. Subsequently, they were transferred to a separate euthanasia chamber and exposed to 80% carbon dioxide for terminal procedures. Death was confirmed by cessation of spontaneous breathing and pupillary dilation. The bilateral femora, serum, and colon tissues (1 cm distal to the cecum) [38] were harvested for further analysis.

### 2.4. Treadmill Exercise Intervention Protocol

The treadmill exercise intervention protocol was adapted and modified based on previous reports [39]. The selection of exercise modalities and their parameters is based on the standards of anaerobic (equivalent to 85%–90% of maximum oxygen consumption) and aerobic exercise (equivalent to 65%–70% of maximum oxygen consumption) [39, 40]. Starting from the 4^th^ week post‐OVX surgery, mice in the OVX‐HIIT and OVX‐MICE groups underwent a 3‐day treadmill adaptation training session, lasting 20 min per day. After a 5‐min warm‐up at 4 m/min, the OVX‐HIIT group performed interval training with speeds gradually increasing from 10 to 16 m/min, alternating between 2 min of exercise and 2 min of rest. The OVX‐MICE group, after the warm‐up, performed continuous exercise with speeds gradually increasing from 4 to 8 m/min. The treadmill was set at a 25‐degree incline. Mice were fasted for 30 min before and after exercise. For mice with poor compliance or those falling behind the treadmill belt, gentle stimulation was provided by tapping the treadmill wall to create noise. Daily adaptation training was conducted between 16:00 and 18:00. Starting from the 5^th^ week post‐OVX surgery, mice in the OVX‐HIIT and OVX‐MICE groups underwent a 1‐h treadmill exercise session (Zhenghua Biological Instruments and Equipment Co., Ltd., Anhui, China) per day. The total daily running distance was consistent between the two groups. The intervention lasted for 12 weeks, with 5 days of exercise and 2 days of rest per week. During the intervention period, mice in the Sham and OVX groups were placed on a stationary treadmill at the same time, with all other conditions remaining consistent. The specific weekly exercise protocol, including speed, time, and distance, is detailed in Table S1.

### 2.5. Micro‐CT Analysis of Femoral Structure

The femoral structure of mice was evaluated using a micro‐CT scanner (SkyScan 1176, Bruker, Karlsruhe, Germany). The scanning parameters were set at 70 kV voltage, 200 μA current, and 18 μm resolution, with a 0.5 mm aluminum filter employed to minimize beam hardening effects. Image reconstruction was performed using NRecon software (Bruker, Karlsruhe, Germany), followed by three‐dimensional (3D) model visualization through CTVox software. For quantitative morphological analysis, a region of interest (ROI) was selected comprising 50 consecutive 0.5 mm slices distal to the growth plate, with subsequent analysis of 100 trabecular bone sections of 2 mm height. Structural parameters of the femoral region were measured using CTAn software (Bruker, Karlsruhe, Germany), including bone mineral density (BMD, g/cm^3^), bone volume/total volume ratio (BV/TV, %), bone surface/total volume ratio (BS/TV, 1/mm), trabecular number (Tb.N, 1/mm), trabecular separation (Tb.Sp, mm), and trabecular thickness (Tb.Th, mm).

### 2.6. Histological and Histomorphometric Analysis

Femoral and colonic tissues from each group were fixed in 4% paraformaldehyde (Servicebio, Wuhan, Hubei, China). After 48 h of fixation, femoral bones were decalcified in 12.5% ethylenediaminetetraacetic acid (EDTA, Servicebio, Wuhan, Hubei, China) for 4 weeks. Both colonic tissues (fixed for 48 h) and decalcified femoral tissues were embedded in paraffin and sectioned at 5 μm thickness. Hematoxylin and eosin (H&E) staining was performed for histological examination of femoral and colonic tissues. Additionally, tartrate‐resistant acid phosphatase (TRAP) staining was conducted to analyze osteoclast formation in femoral tissues. Quantitative assessment of TRAP‐positive osteoclast numbers was performed using Image J software (National Institutes of Health, Bethesda, MD, USA).

### 2.7. Immunohistochemistry (IHC) Analysis

For IHC analysis, femoral and colonic tissue sections were initially equilibrated in 0.1 M tris‐buffered saline for 10 min. Subsequently, sections were blocked with phosphate‐buffered saline (PBS) containing 10% normal goat serum for 1 h at room temperature. Following blocking, femoral sections were incubated overnight at 4°C with the following primary antibodies: osteocalcin (OCN, 1:50), runt‐related transcription factor 2 (RUNX2, 1:100), RANKL (1:200), TNF‐α (1:200), IL‐6 (1:200), and IL‐1β (1:200). Intestinal sections were incubated with primary antibodies against tight junction proteins (TJPs), including ZO‐1 (1:500), occludin (1:500), and claudin‐1 (1:500) under the same conditions.

After primary antibody incubation, sections were washed with PBS for 15 min and then incubated with horseradish peroxidase (HRP)‐conjugated secondary antibody (1:500) for 1 h at room temperature. Immunoreactivity was visualized using 3,3′‐diaminobenzidine (DAB) chromogen for detection of OCN, RUNX2, RANKL, ZO‐1, occludin, claudin‐1, IL‐6, IL‐1β, and TNF‐α expression. Quantitative analysis of positive signals was performed using Image J software (National Institutes of Health, Bethesda, MD, USA) by examining six randomly selected high‐power fields (HPFs) per section.

### 2.8. Enzyme‐Linked Immunosorbent Assay (ELISA)

Serum concentrations of 17 β‐estradiol (E2), along with the expression levels of IL‐6, IL‐1β, and TNF‐α, were quantified using commercial ELISA kits according to the manufacturer’s protocols (eBioscience, Shanghai, China).

### 2.9. Western Blot (WB) Analysis

Colonic tissue sections from each group of mice were collected and homogenized in lysis buffer (Beyotime, Shanghai, China), followed by lysis for 1 h to extract total protein. The samples were then centrifuged at 12,000 rpm for 10 min at 4°C, and the supernatant was collected. The protein concentration in the supernatant was determined using a protein quantification kit (Beyotime, Shanghai, China), and the supernatant was mixed with 5× loading buffer, followed by heating at 100°C for 10 min to denature the proteins. Next, equal amounts of protein were separated by 12% sodium dodecyl sulfate‐polyacrylamide gel electrophoresis (SDS–PAGE), and the separated proteins were transferred to a polyvinylidene fluoride (PVDF) membrane. After transfer, the membrane was blocked with 5% skimmed milk in tris‐buffered saline with 0.05% Tween‐20 (TBST) for 1 h. The membrane was then incubated overnight at 4°C with the following primary antibodies: anti‐ZO‐1 (1:500), anti‐occludin (1:500), anti‐claudin‐1 (1:500), anti‐IL‐6 (1:500), anti‐IL‐1β (1:500), and anti‐TNF‐α (1:500). β‐Actin (1:5000) was used as an internal control. Subsequently, the membrane was washed three times with TBST, each for 10 min, and incubated with HRP‐conjugated secondary antibody (1:10000) at room temperature for 2 h. Finally, protein bands were detected using an enhanced chemiluminescence (ECL) detection kit (Millipore‐Sigma, Burlington, MA, USA), and the protein bands were visualized and analyzed for relative intensity using ImageJ software (National Institutes of Health, Bethesda, MD, USA). The detailed information of antibodies used is provided in Table S2.

### 2.10. Fecal 16S rRNA Sequencing and Bioinformatics Analysis

The DNA from fecal samples was extracted using the e.z.n.a. Fecal DNA Kit (Omega Bio‐tek, Norcross, GA, USA) following the manufacturer’s guidelines. The extracted DNA was then amplified via polymerase chain reaction (PCR) with primers specific for the bacterial 16S V3–V4 rRNA region (V3:341F, CCTACGGGNGGCWGCAG; V4:806R, GGACTACHVGGGTATCTAAT). The resulting amplicons were separated via 2% agarose gel electrophoresis and purified using the AxyPrep DNA Gel Extraction Kit (Axygen Biosciences, Union City, CA, USA), in accordance with the manufacturer’s instructions. The purified PCR products were quantified using a Qubit 3.0 Fluorometer (Life Technologies, Invitrogen), and on average, 20 different barcoded amplicons were pooled for subsequent steps. Illumina paired‐end sequencing libraries were constructed following the Illumina genomic DNA library preparation protocol, and sequencing was performed on the Illumina NovaSeq 6000 platform (Nanjing GenePioneer Co. Ltd) with a standard 2 × 250 paired‐end sequencing protocol.

The microbiome data analysis was carried out by GenePioneer Co. Ltd (Nanjing, Jiangsu, China). The raw paired‐end reads were first demultiplexed based on their unique barcodes (allowing zero mismatches) and primers (allowing upto two mismatches). The reads were then merged into single sequences using PANDAseq (version 2.11) with a minimum overlap of 10 bp and a maximum mismatch ratio of 0.2 in the overlap region. Quality control was rigorously performed using PRINSEQ (version 0.20.4) to filter out sequences with an average quality score below 20 and those containing ambiguous bases (N) exceeding 5% of the total sequence length. Chimera removal was subsequently conducted to eliminate spurious sequences. Following quality filtering, the high‐quality sequences were clustered into amplicon sequence variants (ASVs) using DADA2 within the QIIME2 (version 2021.11) pipeline [41]. Taxonomic assignment was performed using a pre‐trained naive Bayes classifier based on the SILVA reference database. Downstream analyses were performed on the resulting ASV table. Alpha diversity indices and beta diversity metrics were calculated. Principal coordinates analysis (PCoA) and nonmetric multidimensional scaling (NMDS) were conducted to visualize community composition differences. Linear discriminant analysis effect size (LEfSe) was used to identify significant differences between the groups [42]. To assess and account for potential batch effects, we ensured that samples from different experimental groups were randomly distributed across sequencing lanes. Furthermore, the homogeneity of group dispersions was checked using beta dispersion tests, and PERMANOVA was applied to model and test the significance of group differences while considering the overall data structure. PICRUSt2 was used to predict microbial metabolic pathways based on the Kyoto Encyclopedia of Genes and Genomes (KEGG) database. Statistical comparisons were performed using the Mann–Whitney *U* test for two‐group comparisons and the Kruskal–Wallis test for multiple‐group comparisons, with a significance level set at a *p*‐value of <0.05.

### 2.11. Statistical Analysis

Data analysis in this study was performed using GraphPad Prism 9.5 software (GraphPad, San Diego, CA, USA). The normality of the data distribution for each group was assessed using the Kolmogorov–Smirnov test. Based on the normality test results, data are presented as mean ± standard deviation (SD) for normally distributed data, or as median (interquartile range, Q1–Q3) for non‐normally distributed data. For comparisons among multiple groups with normal distribution, one‐way analysis of variance (ANOVA) was employed. To control the family‐wise error rate for multiple comparisons, Tukey’s honestly significant difference (HSD) post hoc test was applied following a significant ANOVA *F*‐test. Data that did not meet the assumption of normality were analyzed using the Kruskal–Wallis test, followed by Dunn’s post hoc test for multiple comparisons. Sham, OVX‐MICE, and OVX‐HIIT groups were statistically compared only against the OVX group. A two‐tailed *p*‐value of <0.05 was considered statistically significant.

## 3. Results

### 3.1. MICE, Rather Than HIIT, Reverses OVX‐Induced Weight Gain and Grip Strength Decline Without Elevating Estrogen Levels

As shown in Figure 1B–D, compared to the Sham group, OVX mice exhibited a significantly higher rate of weight gain and a greater body weight increase at 12 weeks, along with a notable decline in grip strength. Following MICE intervention, the OVX‐MICE group showed a significant reduction in weight gain rate and body weight increase at 12 weeks, as well as a marked improvement in grip strength. However, after HIIT intervention, the OVX‐HIIT group displayed no statistically significant differences in weight gain rate, body weight increase at 12 weeks, or grip strength compared to the OVX group.

As illustrated in Figure 1E, serum E2 levels were significantly lower in the OVX group compared to the Sham group. Neither MICE nor HIIT interventions had any discernible effect on serum E2 levels in OVX mice.

### 3.2. MICE, Rather Than HIIT, Delays OVX‐Induced Bone Microstructure Damage and Bone Mass Loss

To investigate the effects of different exercise intervention modes on OVX‐induced OP, we analyzed the bone mass and bone microstructure of mice in each group using micro‐CT and H&E staining. As shown in Figure 2A,B, compared to the Sham group, OVX mice exhibited significant bone mass loss at the distal femur, a reduction in trabecular bone area, and bone microstructure damage. Quantitative analysis confirmed that OVX significantly compromised key trabecular bone parameters, an effect that was markedly reversed by MICE intervention but not by HIIT (Figure 2C–H). HIIT had no significant effect on these changes, whereas MICE significantly alleviated the alterations in these parameters induced by OVX, indicating that MICE, rather than HIIT, can reverse the bone microstructure damage and bone mass loss induced by OVX.

Figure 2Effect of exercise intervention on the bone loss in mice with OVX‐induced OP. (A) Micro‐computed tomography (micro‐CT) images of femoral tissue. (B) Hematoxylin and eosin (HE) staining with scale bars of 200 μm (upper) and 50 μm (lower). (C–H) Micro‐CT parameters analysis, including bone mineral density (BMD), bone volume per tissue volume (BV/TV), bone surface per tissue volume (BS/TV), trabecular number (Tb.N), trabecular separation (Tb.Sp), and trabecular thickness (Tb.Th). Data represent the mean ± SD, *n* = 8 per group,  ^∗^ *p* < 0.05,  ^∗∗^ *p* < 0.01,  ^∗∗∗^ *p* < 0.001, and  ^∗∗∗∗^ *p* < 0.0001 compared with the corresponding group.

**Figure:**
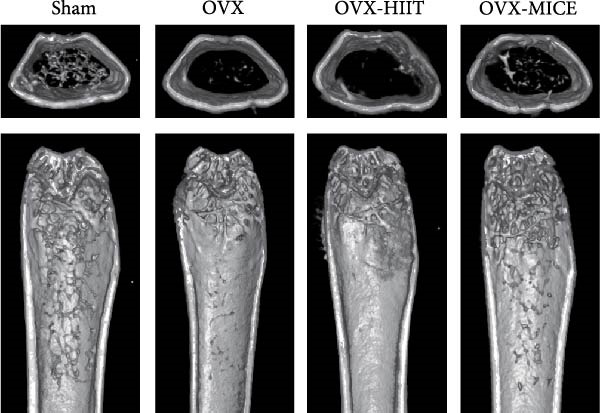
(A)

**Figure:**
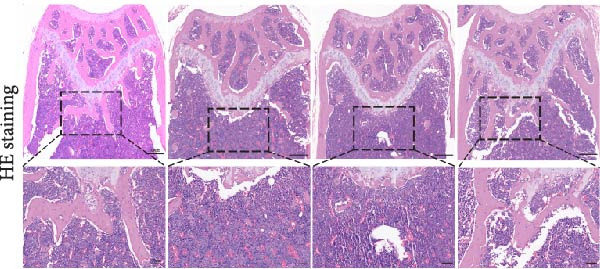
(B)

**Figure:**
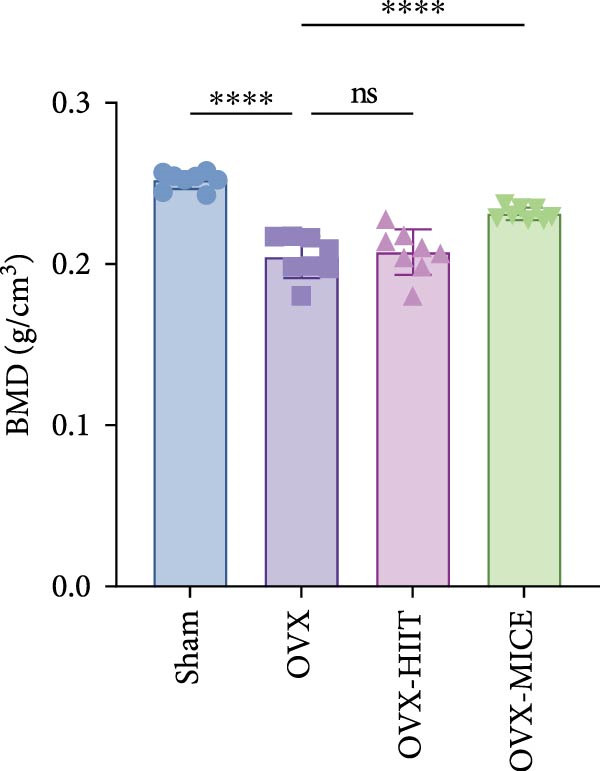
(C)

**Figure:**
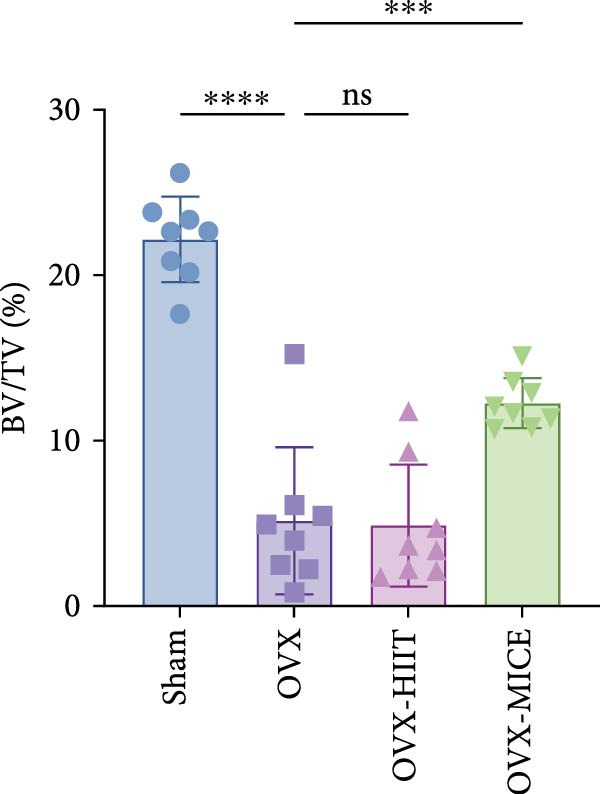
(D)

**Figure:**
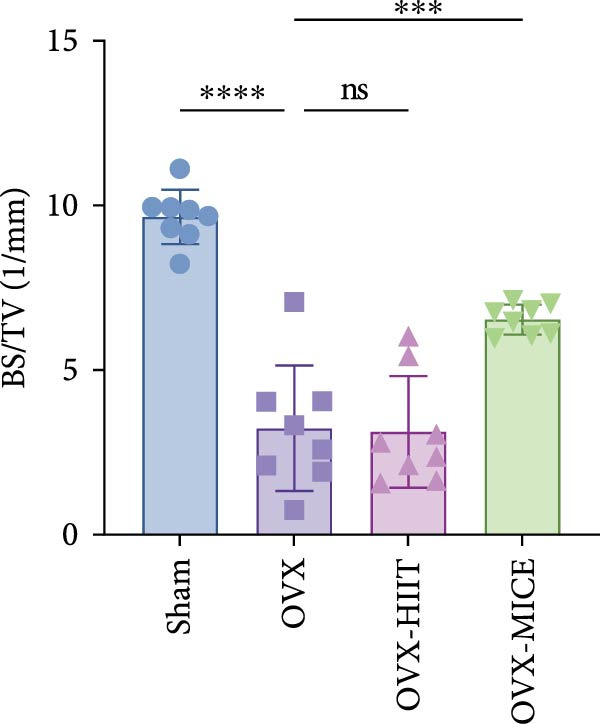
(E)

**Figure:**
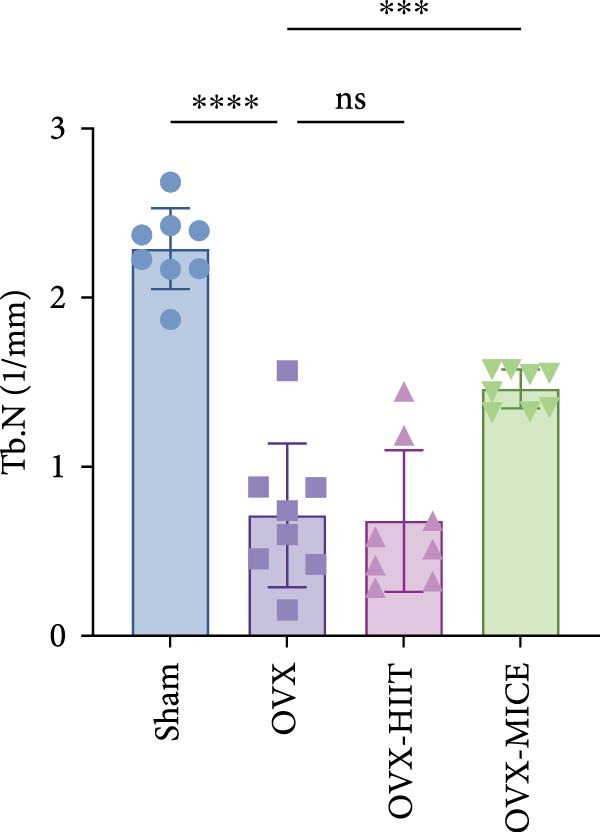
(F)

**Figure:**
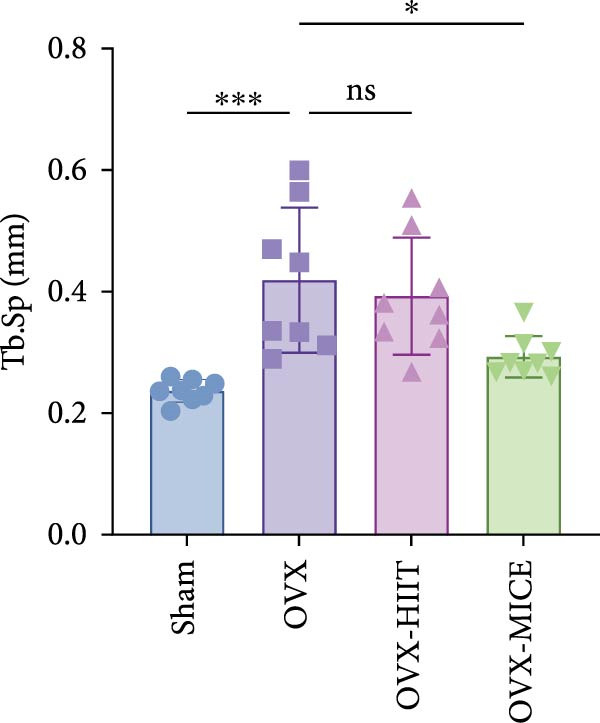
(G)

**Figure:**
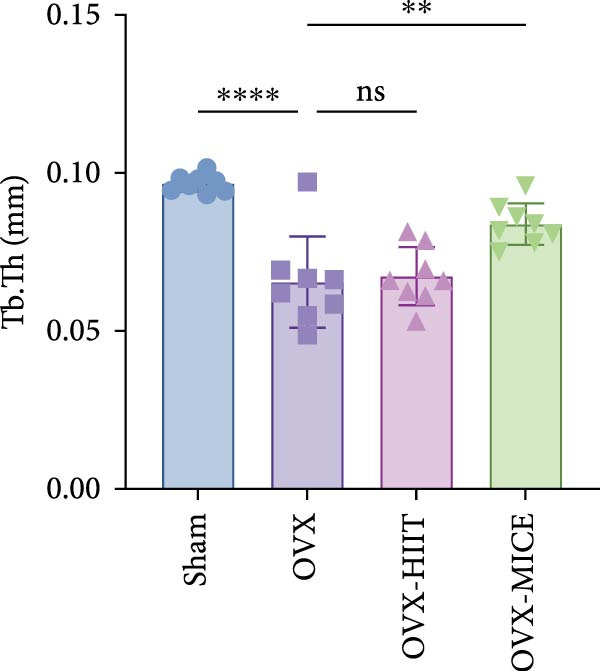
(H)

### 3.3. Both MICE and HIIT Ameliorate OVX‐Induced Osteogenesis Reduction, While Only MICE Attenuates Inflammatory Osteoclastogenic Factor Release and Suppresses OVX‐Induced Osteoclast Enhancement

As shown in Figure 3, OVX significantly reduced the expression of RUNX2 in the growth plate and OCN in the marrow cavity of mice, while increasing the expression of RANKL and the proportion of TRAP^+^ cells, indicating an inhibitory effect on osteoblast activity while promoting osteoclast activation. Both HIIT and MICE showed a trend of promoting the expression of RUNX2 in the growth plate and OCN in the marrow cavity of OVX mice, but the differences were not statistically significant (for IHC score of RUNX2: OVX‐HIIT vs. OVX, *p* = 0.13, OVX‐MICE vs. OVX, *p* = 0.22; for IHC score of OCN: OVX‐HIIT vs. OVX, *p* = 0.22, OVX‐MICE vs. OVX, *p* = 0.36; all *p*  > 0.05, ns). However, MICE significantly inhibited OVX‐induced osteoclast activation, as evidenced by the decrease in RANKL expression and the reduction in the proportion of TRAP^+^ cells. HIIT, on the other hand, showed a trend toward enhanced osteoclast activation, but the differences were not statistically significant (for IHC score of RANKL: OVX‐HIIT vs. OVX, *p* = 0.30; for propotion of TRAP^+^ cell/BS per HPF, OVX‐HIIT vs. OVX, *p* = 0.17, all *p*  > 0.05, ns).

Figure 3Effect of exercise intervention on the expression of osteogenesis and osteoclastogenesis‐related markers in femur of mice with OVX‐induced OP. Representative immunohistochemistry (IHC) staining results of (A) runt‐related transcription factor 2 (RUNX2) and (B) osteocalcin (OCN) IHC staining. (C) Receptor activator of nuclear factor kappa‐B ligand (RANKL) and (D) representative tartrate‐resistant acid phosphatase (TRAP) staining results in femur of each group of mice. Semi‐quantitative analysis of IHC scores for (E) RUNX2, (F) OCN, and (G) RANKL expression in femoral IHC experiments. (H) Analysis of the number of TRAP‐positive osteoclast cells. Data represent the mean ± SD, *n* = 8 per group;  ^∗^ *p* < 0.05,  ^∗∗^ *p* < 0.01,  ^∗∗∗^ *p* < 0.001, and  ^∗∗∗∗^ *p* < 0.0001.

**Figure:**
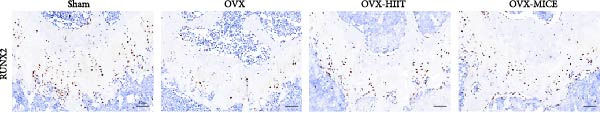
(A)

**Figure:**
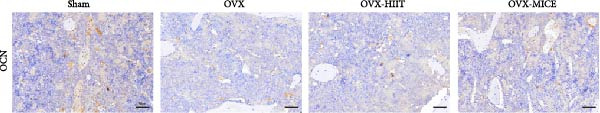
(B)

**Figure:**
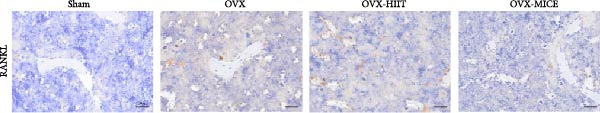
(C)

**Figure:**
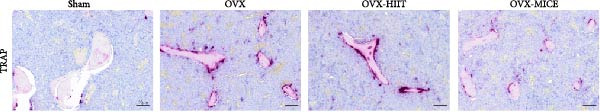
(D)

**Figure:**
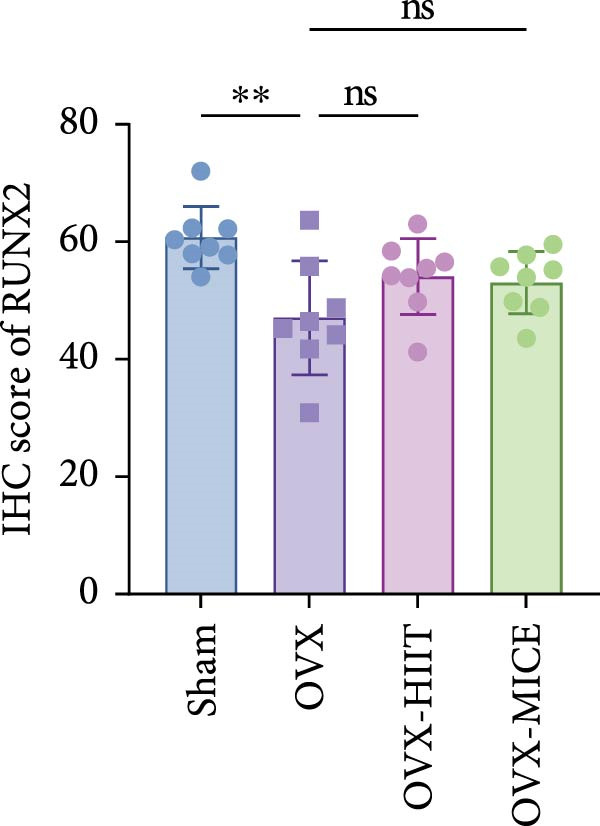
(E)

**Figure:**
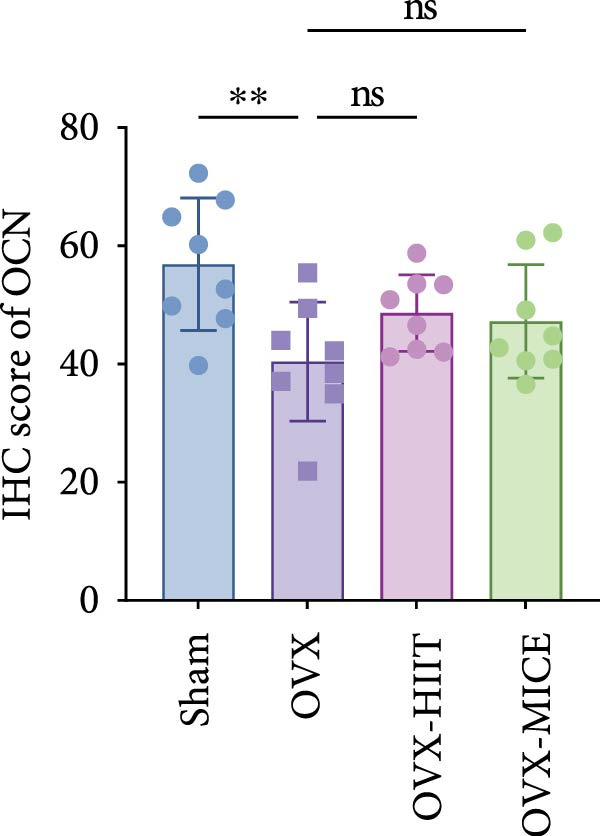
(F)

**Figure:**
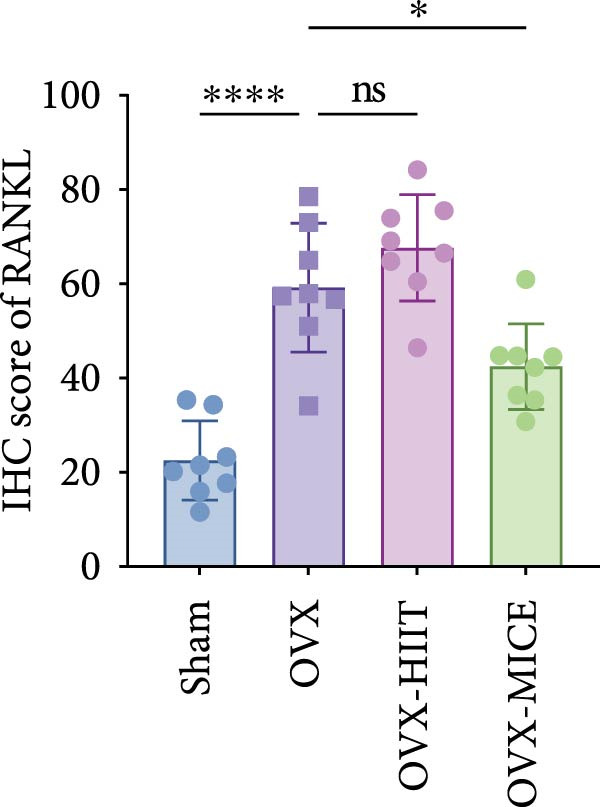
(G)

**Figure:**
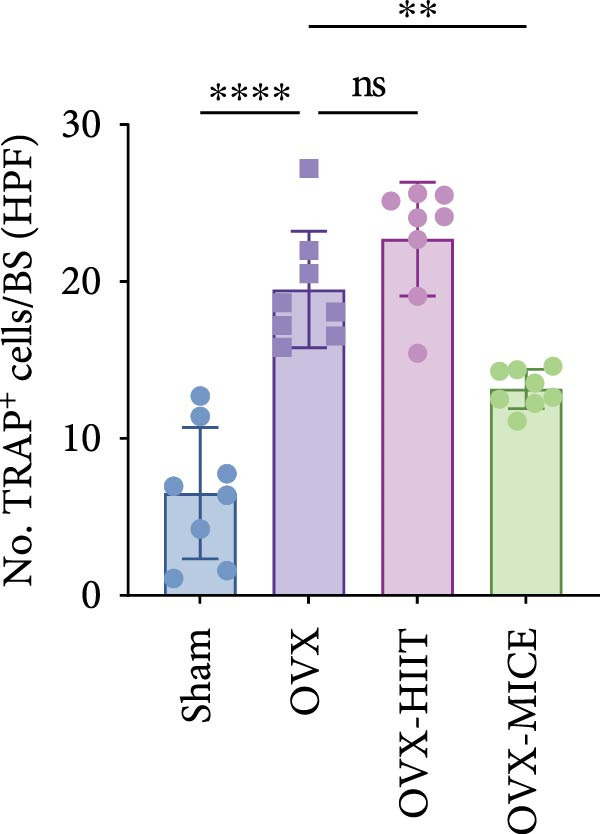
(H)

Based on this, we further evaluated the expression of inflammatory osteoclastogenic factors (IL‐6, IL‐1β, and TNF‐α) in the bone tissue of the mice in each group. As shown in Figure 4A–F, OVX significantly increased the expression of inflammatory osteoclastogenic factors in the femurs of mice, which can be significantly inhibited by MICE, whereas HIIT had no significant effect on these factors. As shown in Figure 4G–I, to further clarify the sources of these inflammatory factors, we assessed the levels of inflammatory osteoclastogenic factors in the serum of each group via ELISA. The results indicated that MICE significantly reduced the serum levels of IL‐6 and IL‐1β in OVX mice, with no evident alternations in TNF‐α (for concentration of serum TNF‐α (pg/mL): OVX‐MICE vs. OVX, *p* = 0.14, ns). After HIIT intervention, there were no significant changes in the serum levels of inflammatory osteoclastogenic factors (IL‐6, IL‐1β, and TNF‐α) in OVX mice, but these levels were significantly higher than those in the OVX‐MICE group.

Figure 4Effect of exercise intervention on femoral and systematic inflammation in mice with OVX‐induced OP. Representative IHC staining results of (A) interleukin‐6 (IL‐6), (B) IL‐1β, and (C) tumor necrosis factor‐alpha (TNF‐α) in the femur of each group of mice. Semi‐quantitative analysis of IHC scores for (D) IL‐6, (E) IL‐1β, and (F) TNF‐α expression in femoral IHC experiments. Serum concentration of inflammatory factors in each group of mice, including (G) IL‐6, (H) IL‐1β, and (I) TNF‐α. Data represent the mean ± SD, *n* = 8 per group;  ^∗^ *p* < 0.05,  ^∗∗^ *p* < 0.01,  ^∗∗∗^ *p* < 0.001, and  ^∗∗∗∗^ *p* < 0.0001.

**Figure:**
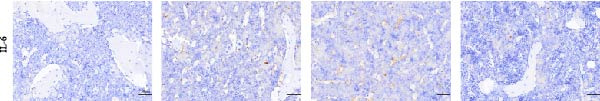
(A)

**Figure:**
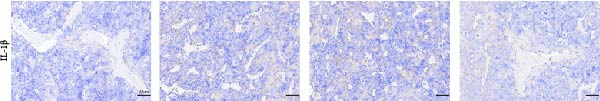
(B)

**Figure:**
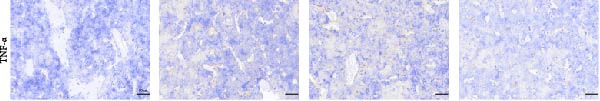
(C)

**Figure:**
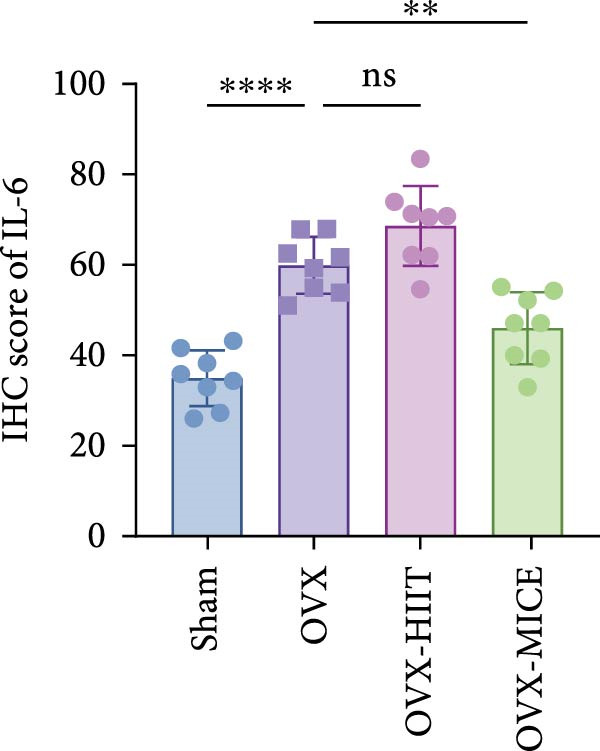
(D)

**Figure:**
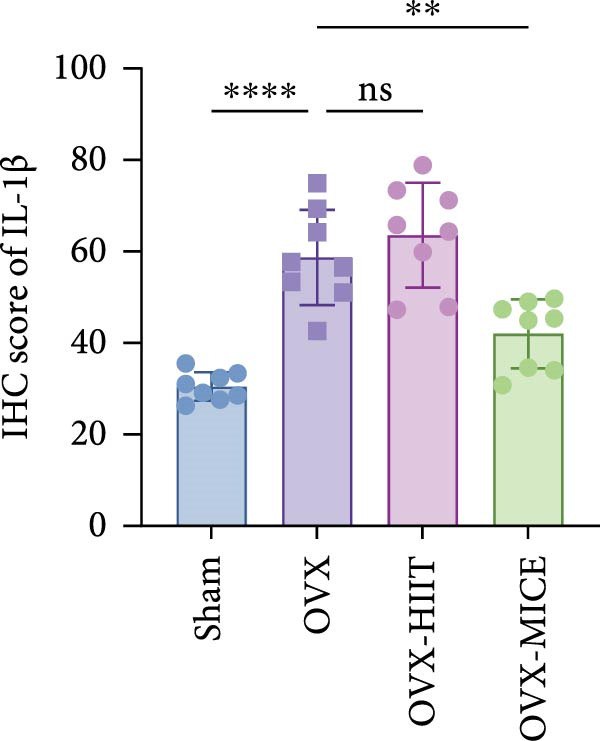
(E)

**Figure:**
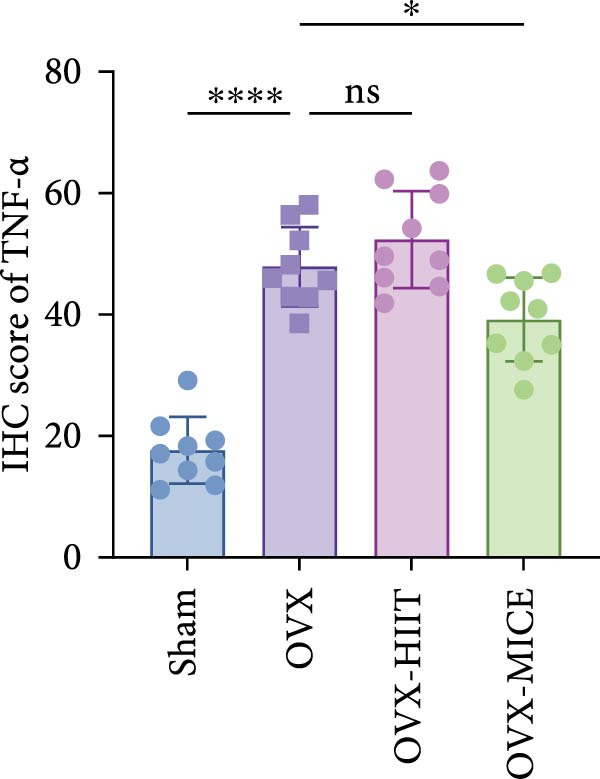
(F)

**Figure:**
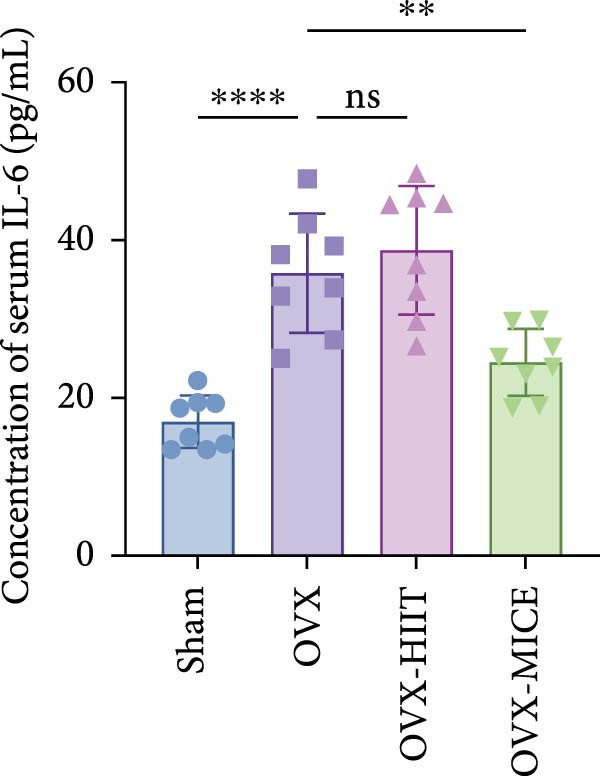
(G)

**Figure:**
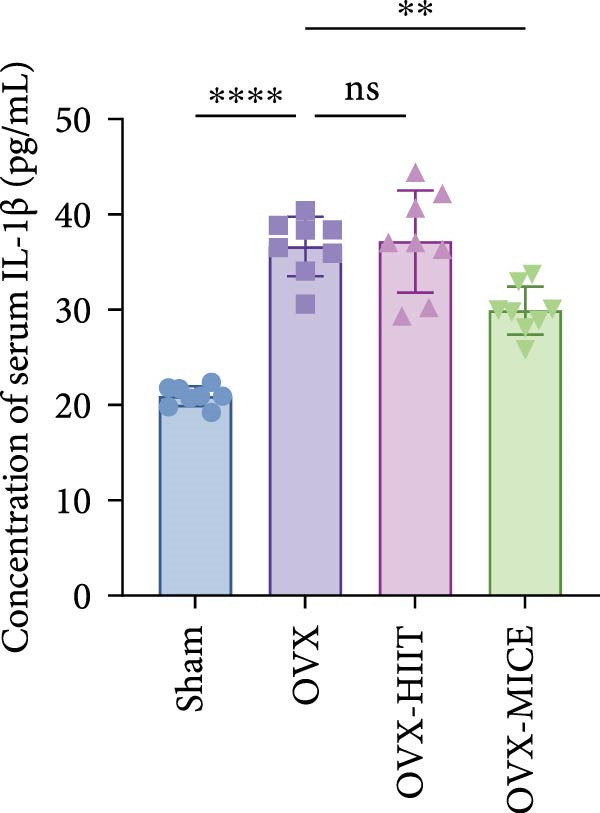
(H)

**Figure:**
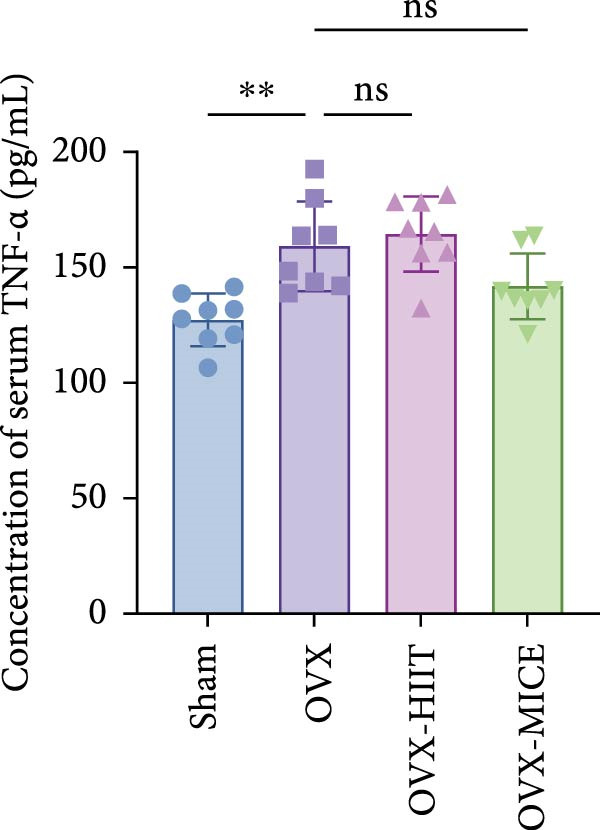
(I)

In summary, these results suggest that different exercise modalities have distinct effects on OVX‐induced OP. MICE can protect against bone microstructure damage and bone mass loss induced by OVX. This may be due to MICE’s role in regulating bone metabolism by inhibiting the expression of osteoclastogenic factors in circulation and the femur, thereby reducing bone resorption. In contrast, HIIT has no significant effect on this process.

### 3.4. MICE, Rather Than HIIT, Reversed the OVX‐Mediated Disruption of the Intestinal Mucosal Barrier in Mice

To further clarify the sources of inflammatory osteoclastogenic factors in circulation and femur, and based on the “gut–bone” axis theory, we investigated the changes in the colons of OVX‐induce OP mice under different exercise intervention models. As shown in Figure 5A, H&E staining of the colons revealed that, compared to the Sham group, OVX mice had a sparser intestinal lumen and larger inter‐wall gaps, indicating that estrogen deficiency induced by OVX was associated with damage to the intestinal mucosal barrier. MICE intervention improved these changes, whereas HIIT intervention did not exhibit a significant effect. Furthermore, as shown in Figure 5B–K, IHC and WB analyses revealed that, compared to the Sham group, OVX mice had significantly reduced levels of TJPs such as ZO‐1, occludin, and claudin‐1 in the intestinal wall, suggesting damage to the intestinal mucosal barrier and increased intestinal permeability. These markers significantly increased after MICE intervention, whereas HIIT had no noticeable effect. These results indicate that OVX can mediate the disruption of the intestinal epithelial barrier and increase intestinal permeability in mice, while MICE intervention can restore the integrity of the intestinal epithelial barrier and reduce intestinal permeability, with no significant effect observed with HIIT.

Figure 5Effect of exercise intervention on integrity of intestinal barrier in the mice with OVX‐induced OP. (A) Representative HE staining results of the colon in each group of mice. Representative IHC staining results of (B) zonula occludens‐1 (ZO‐1), (C) occludin, and (D) claudin‐1 in the colon of each group of mice. Semi‐quantitative analysis of IHC scores for (E) ZO‐1, (F) occludin, and (G) claudin‐1 expression in the colon of each group of mice. (H) Western blot (WB) results of ZO‐1, occludin, and claudin‐1 expression in the colon of mice. Semi‐quantitative analysis of (I) ZO‐1, (J) occludin, and (K) claudin‐1 protein expression in the colon of mice. Data represent the mean ± SD, *n* = 8 per group;  ^∗^ *p* < 0.05,  ^∗∗^ *p* < 0.01,  ^∗∗∗^ *p* < 0.001, and  ^∗∗∗∗^ *p* < 0.0001.

**Figure:**
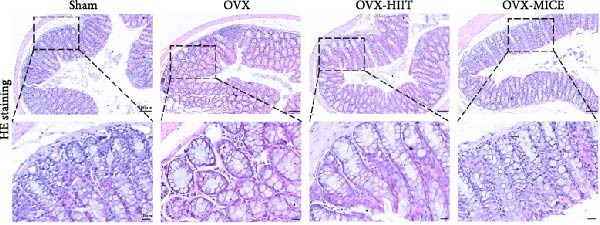
(A)

**Figure:**
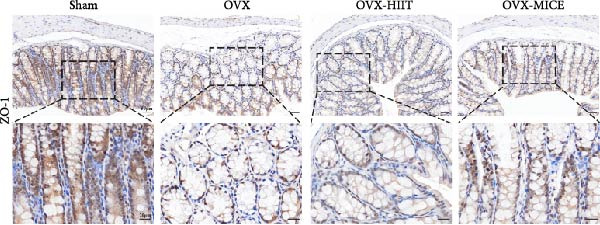
(B)

**Figure:**
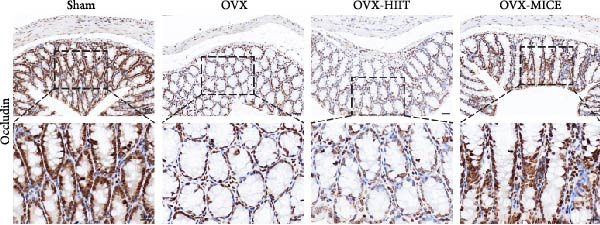
(C)

**Figure:**
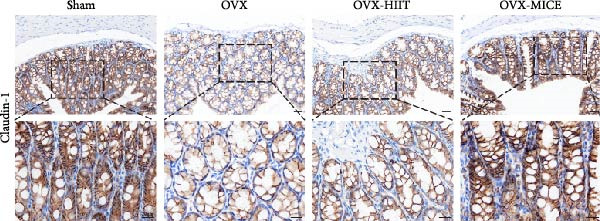
(D)

**Figure:**
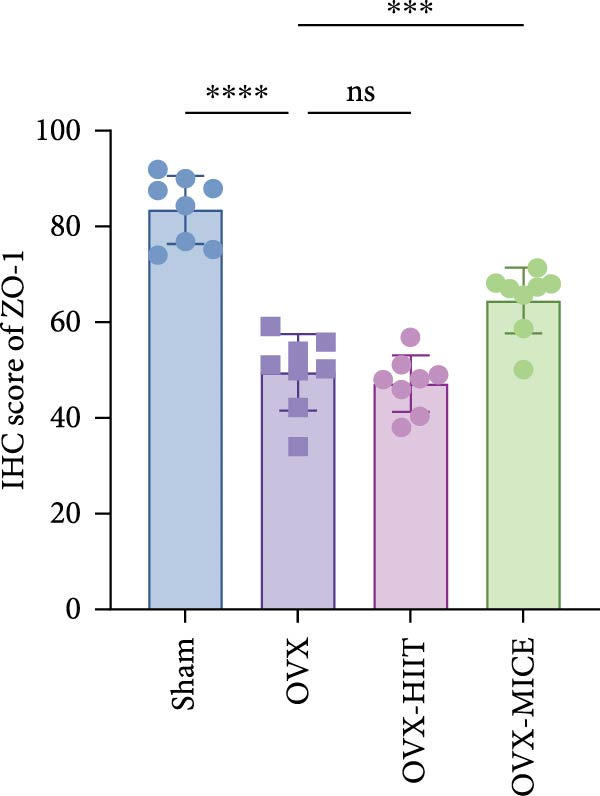
(E)

**Figure:**
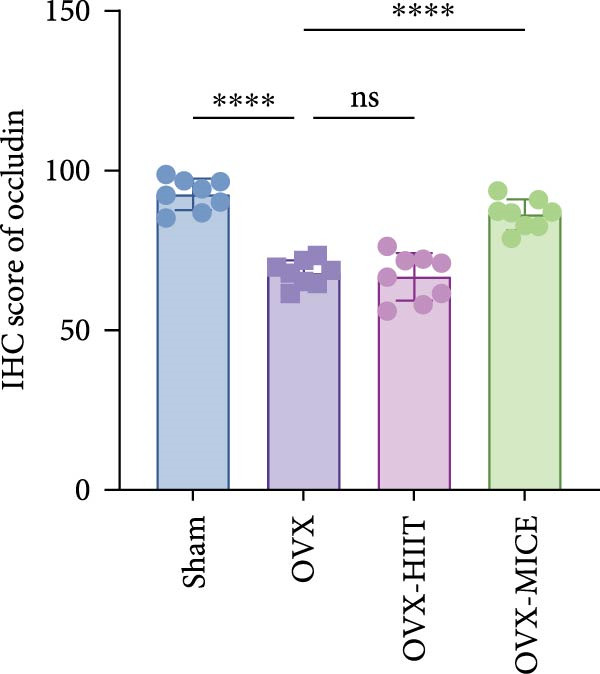
(F)

**Figure:**
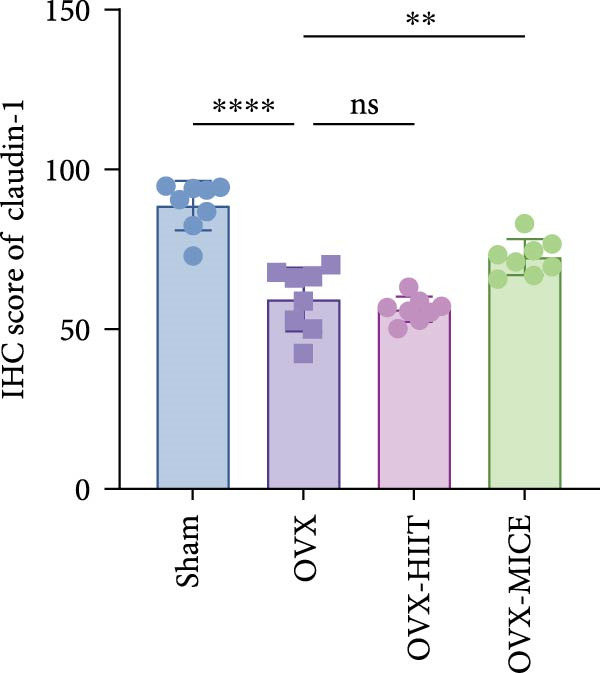
(G)

**Figure:**
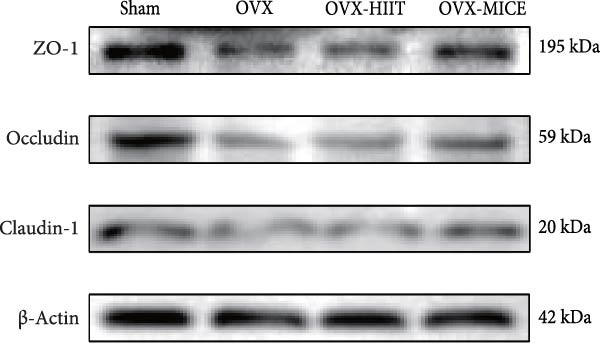
(H)

**Figure:**
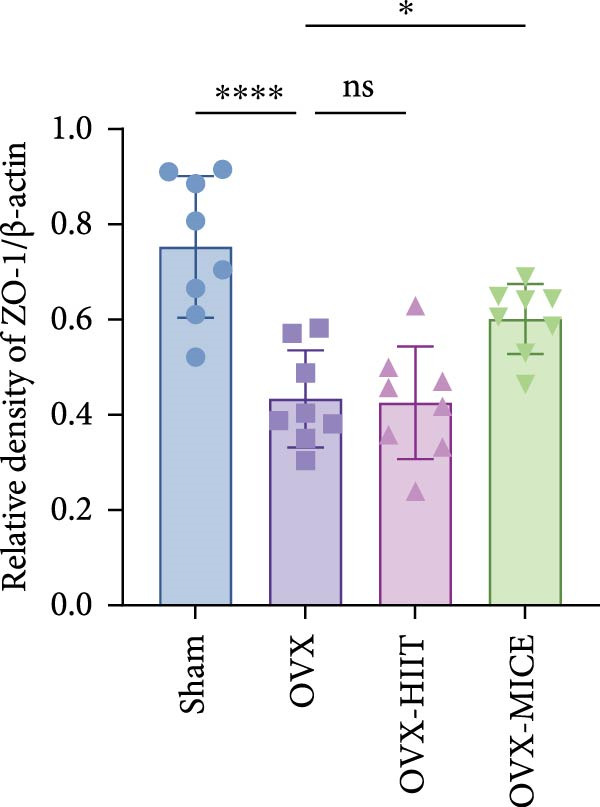
(I)

**Figure:**
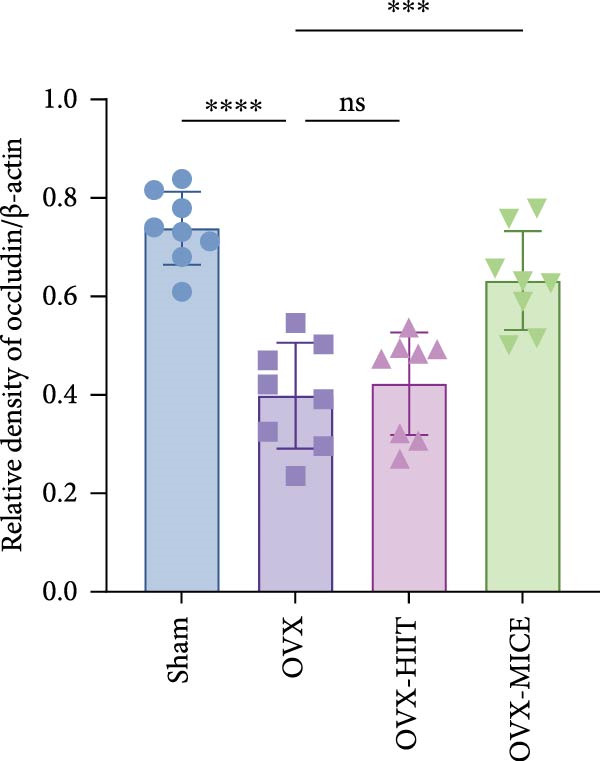
(J)

**Figure:**
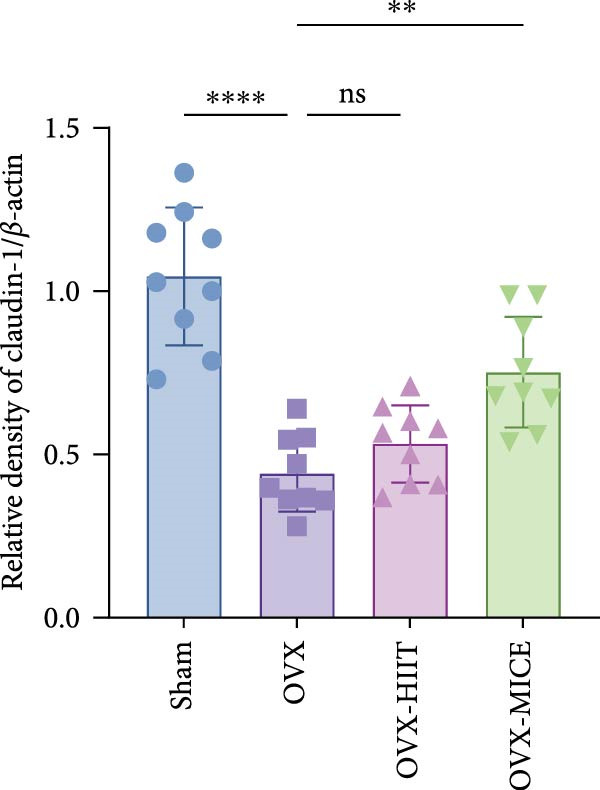
(K)

### 3.5. MICE, but Not HIIT, Significantly Attenuates OVX‐Induced Release of Pro‐Osteoclastogenic Inflammatory Factors in the Intestinal Tract

To further evaluate the expression of pro‐osteoclastogenic inflammatory factors (IL‐6, IL‐1β, and TNF‐α) in colonic tissues, immunohistochemistry (IHC) and WB analyses were performed (Figure 6). The results revealed that OVX significantly upregulated the expression of IL‐6, IL‐1β, and TNF‐α in colonic tissues compared to the Sham group, while MICE intervention markedly suppressed OVX‐induced increase of pro‐osteoclastogenic inflammatory factors, whereas HIIT exhibited no such protective effect. Notably, HIIT even exacerbated colonic inflammation, as evidenced by significantly higher TNF‐α expression in the OVX‐HIIT group compared to the OVX group (Figure 6G and J). These findings suggest that MICE effectively counteract OVX‐driven elevation of pro‐osteoclastogenic inflammatory factors in the colon, while HIIT may potentiate intestinal inflammatory responses, aligning with observations in serum and femoral tissues.

Figure 6Effect of exercise intervention on the intestinal inflammation in mice with OVX‐induced OP. Representative IHC staining results of (A) IL‐6, (B) IL‐1β, and (C) TNF‐α in the colon of each group of mice. Semi‐quantitative analysis of IHC scores for (D) IL‐6, (E) IL‐1β, and (F) TNF‐α expression in the colon of mice. (G) WB results of IL‐6, IL‐1β, and TNF‐α in the colon of mice. Semi‐quantitative analysis of (H) IL‐6, (I) IL‐1β, and (J) TNF‐α protein expression in the colon of mice. Data represent the mean ± SD, *n* = 8 per group;  ^∗^ *p* < 0.05,  ^∗∗^ *p* < 0.01,  ^∗∗∗^ *p* < 0.001, and  ^∗∗∗∗^ *p* < 0.0001.

**Figure:**
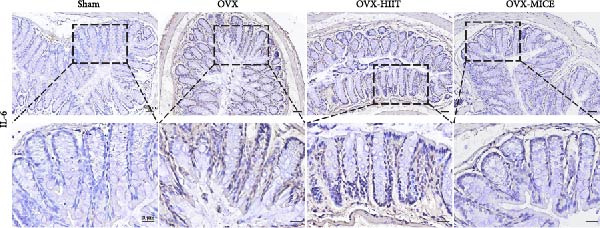
(A)

**Figure:**
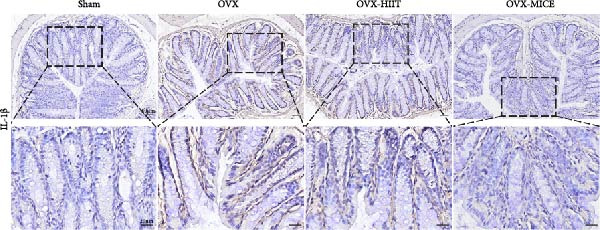
(B)

**Figure:**
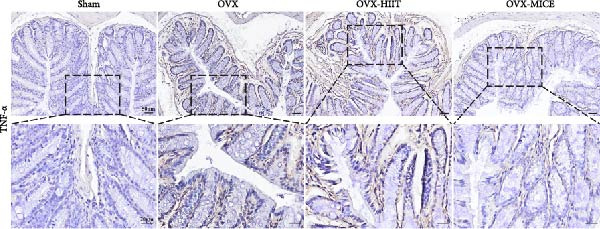
(C)

**Figure:**
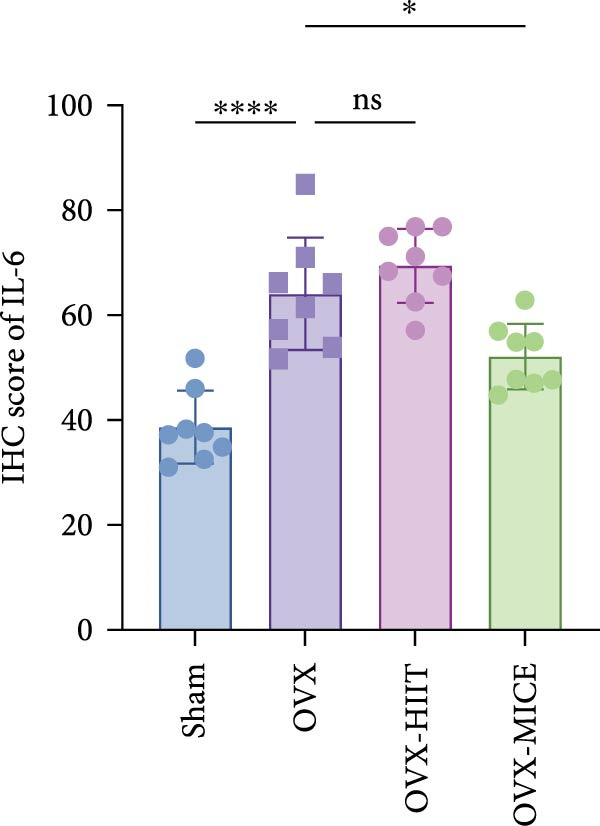
(D)

**Figure:**
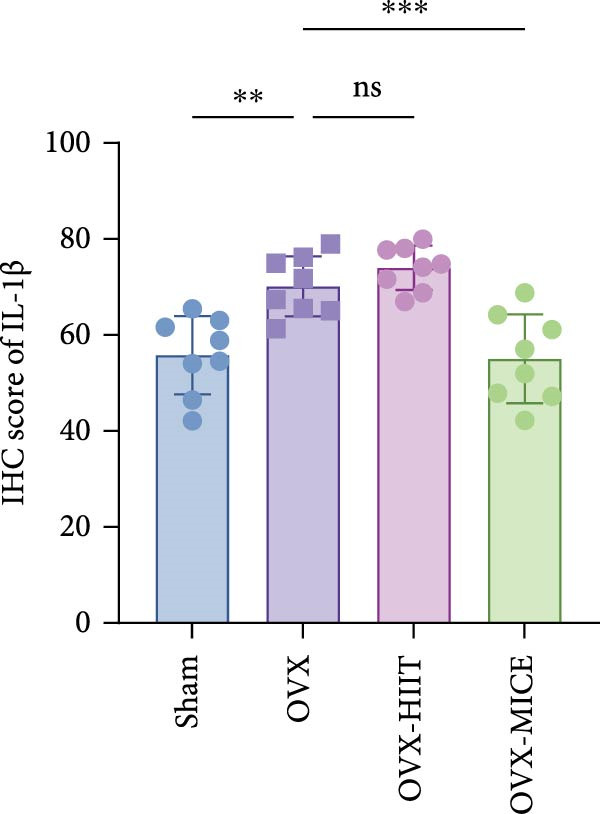
(E)

**Figure:**
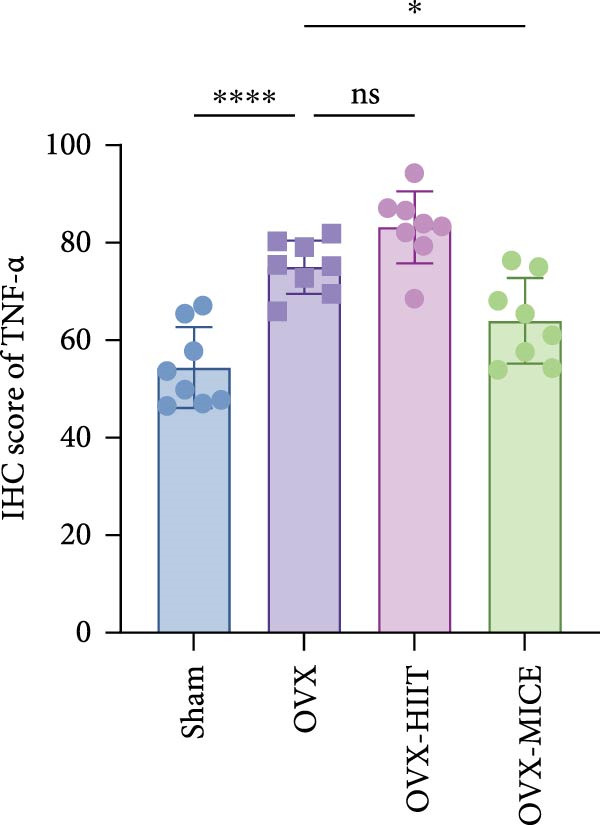
(F)

**Figure:**
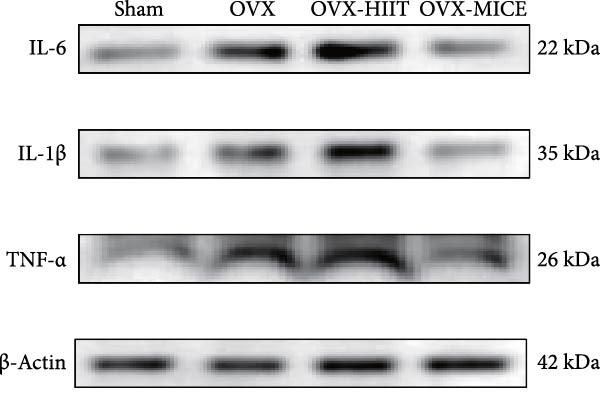
(G)

**Figure:**
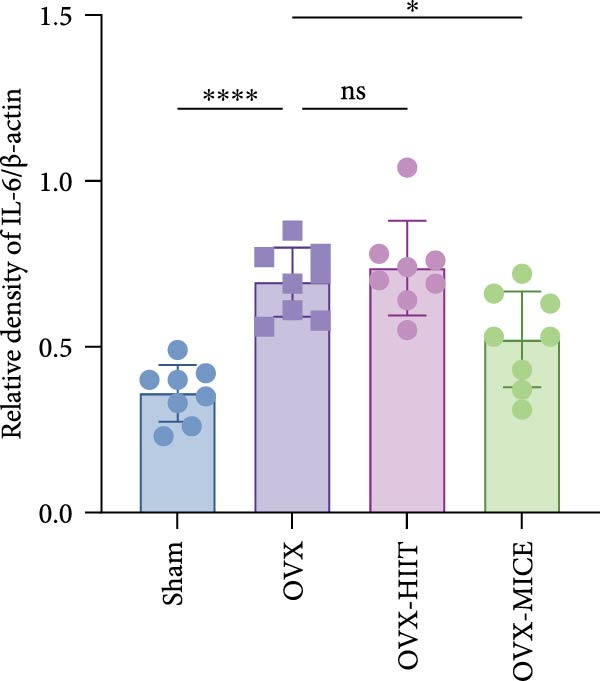
(H)

**Figure:**
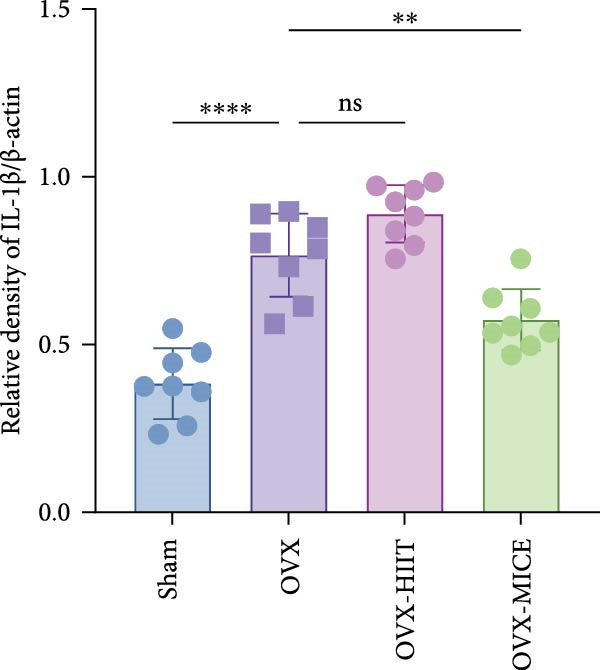
(I)

**Figure:**
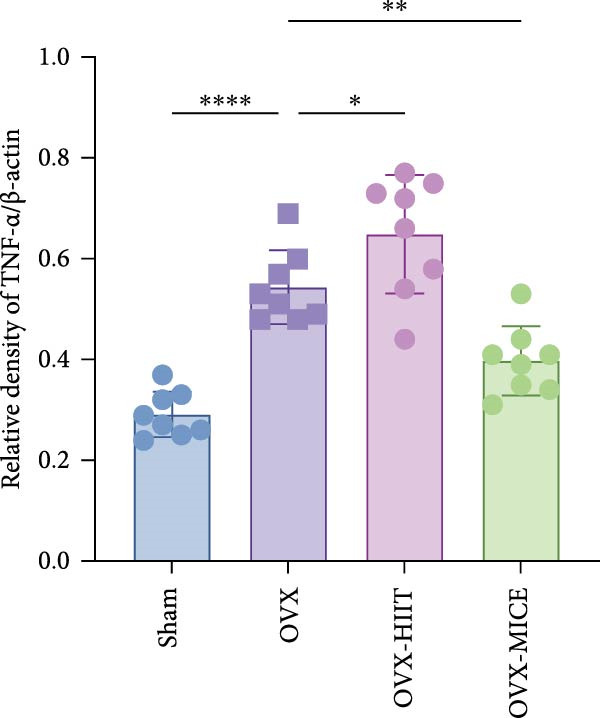
(J)

### 3.6. MICE Ameliorates OVX‐Induced GM Dysbiosis

Given the critical role of GM dysbiosis in intestinal barrier disruption and systemic inflammation [43], we analyzed GM composition and diversity via 16S rRNA V3–V4 sequencing. In α‐diversity assessments (Figure 7A–F), the OVX group exhibited significantly reduced microbial α‐diversity across multiple indices compared to the Sham group. MICE intervention restored these indices, demonstrating enhanced microbial richness, evenness, and phylogenetic diversity in OVX mice. In contrast, HIIT failed to improve the α‐diversity impairment induced by OVX. β‐Diversity analysis (Figure 7G,H) through PCoA and NMDS revealed distinct clustering patterns: OVX mice displayed divergent GM profiles from Sham controls, with reduced community aggregation. MICE restored microbial community cohesion in OVX mice, whereas HIIT further dispersed GM distribution. At the phylum level (Figure 7I), OVX increased the Firmicutes/Bacteroidota (F/B) ratio, a hallmark of dysbiosis. MICE normalized this ratio, while HIIT showed no regulatory effect (Figure 7J). Distribution histogram of LDA values and LEfSe analysis of phylogenetic tree (Figure 7K,L) identified taxa significantly enriched in the OVX‐MICE group, including f_Prevotellaceae, g_Eubacterium_coprostanoligenes_group, s_Muribaculum intestinale, and g_Blautia. Collectively, these data demonstrate that MICE, but not HIIT, mitigates OVX‐induced GM dysbiosis.

Figure 7Effect of exercise intervention on the imbalance of gut microbiota (GM) in mice with OVX‐induced OP. (A) ACE, (B) Chao1, (C) Shannon, (D) Simpson, (E) Faith_pd, and (F) Observed_features indices. (G) Principal coordinate analysis (PCoA) analysis of gut microbiota composition. (H) Nonmetric multidimensional scaling (NMDS) analysis of gut microbiota composition. (I) Phylum‐level microbial community abundance. (J) The ratio of Firmicutes and Bacteroidetes phyla in the gut microbiota of each group of mice. (K) Distribution histogram of linear discriminant analysis (LDA) values. (L) LDA Effect Size (LEfSe) analysis of phylogenetic tree. Data represent the mean ± SD, *n* = 8 per group;  ^∗^ *p* < 0.05,  ^∗∗^ *p* < 0.01,  ^∗∗∗^ *p* < 0.001, and  ^∗∗∗∗^ *p* < 0.0001.

**Figure:**
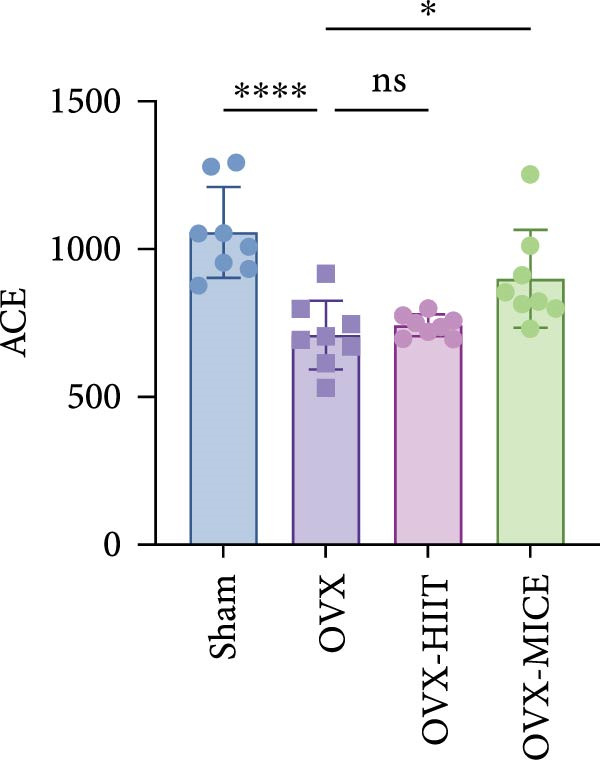
(A)

**Figure:**
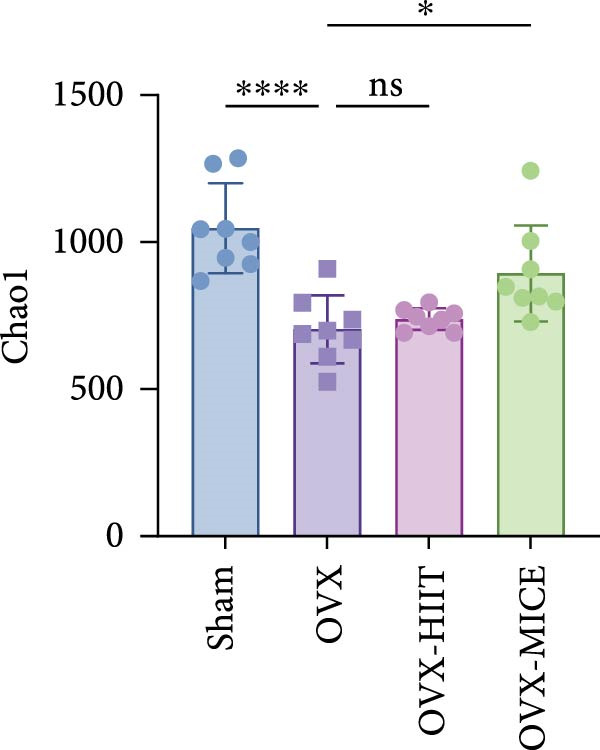
(B)

**Figure:**
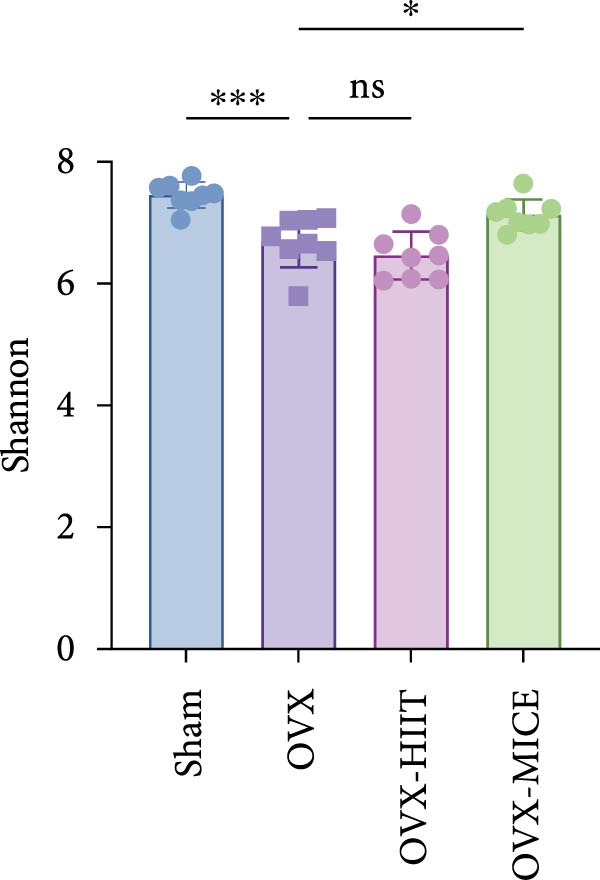
(C)

**Figure:**
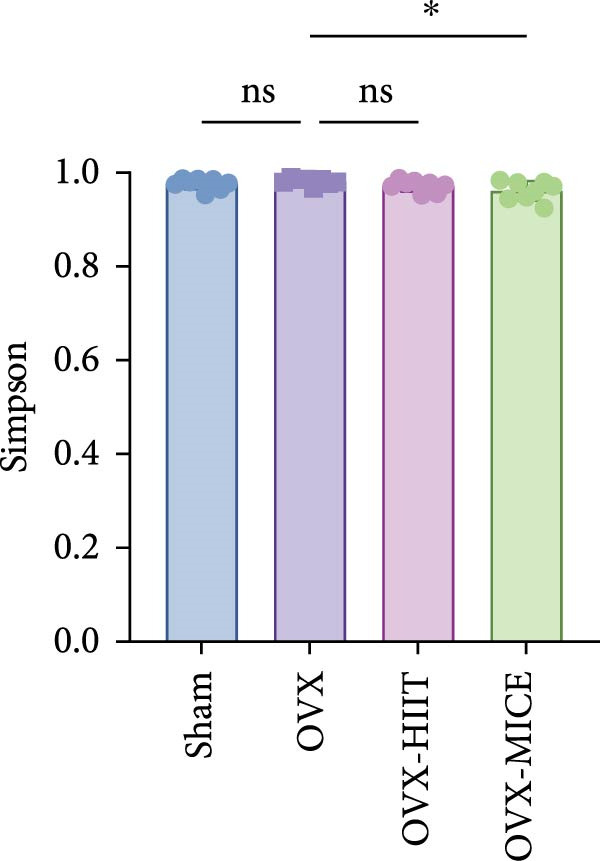
(D)

**Figure:**
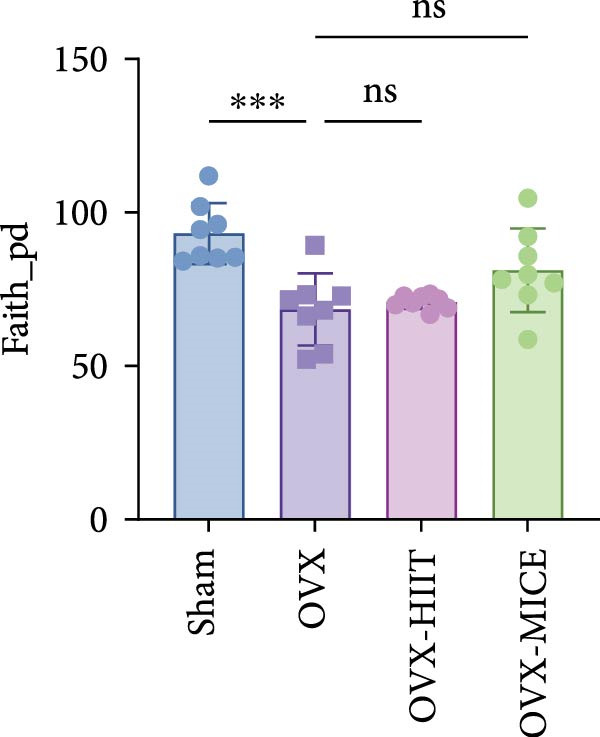
(E)

**Figure:**
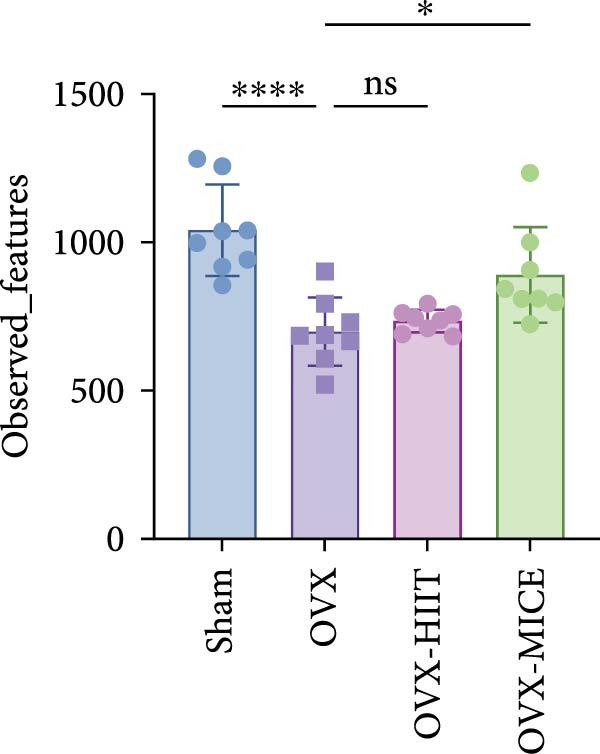
(F)

**Figure:**
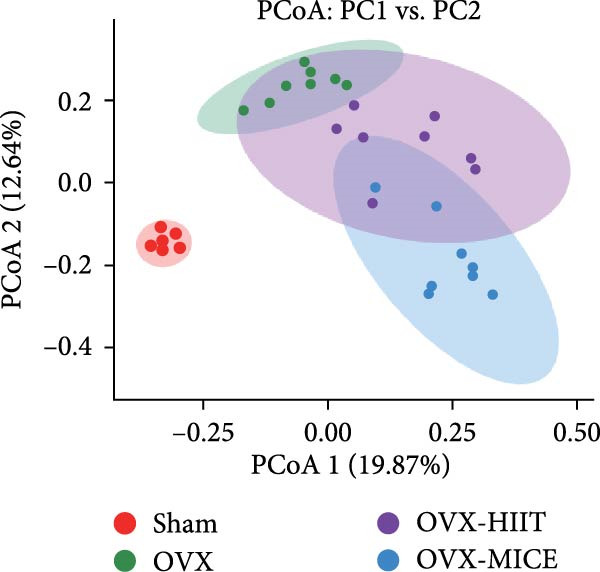
(G)

**Figure:**
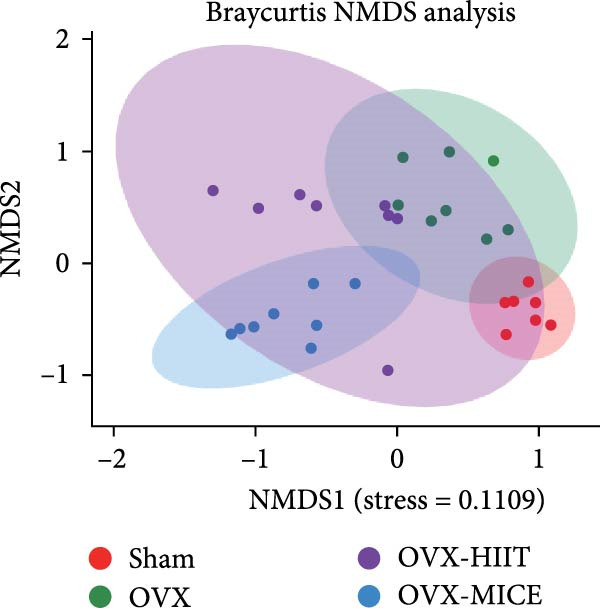
(H)

**Figure:**
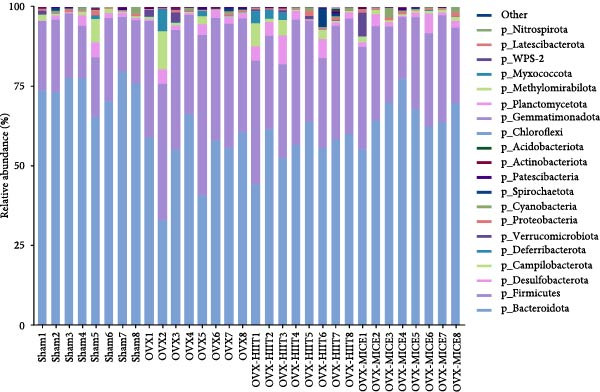
(I)

**Figure:**
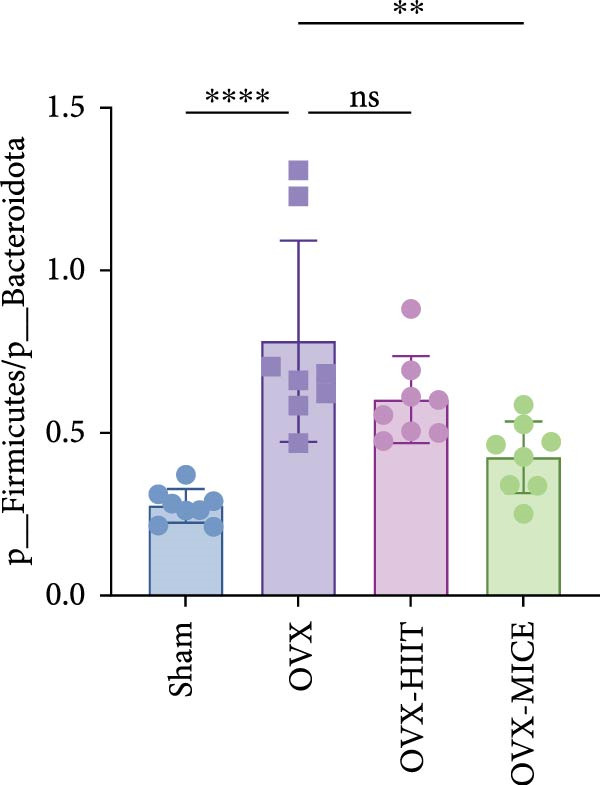
(J)

**Figure:**
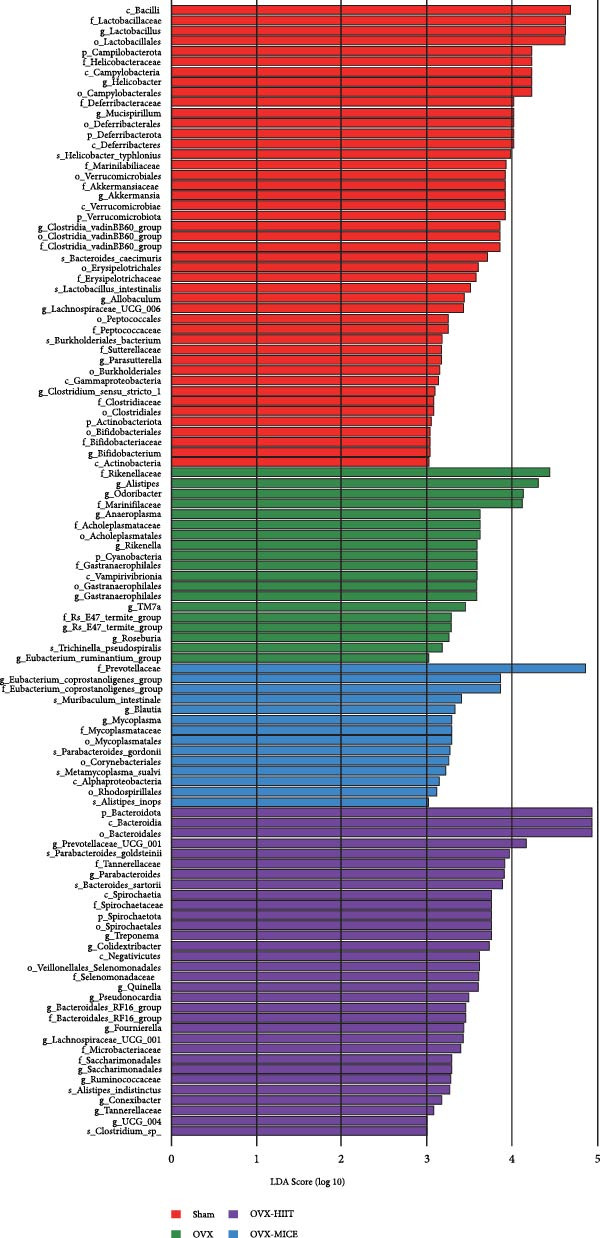
(K)

**Figure:**
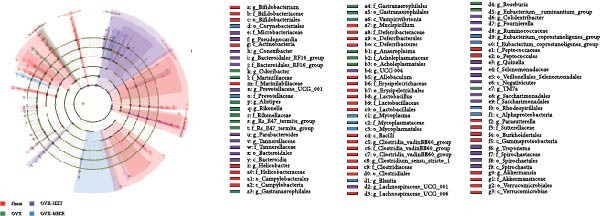
(L)

### 3.7. The Prediction of Potential Metabolic Functions of GM

PICRUSt‐based functional prediction (Figure 8A,B) revealed substantial differences in KEGG pathway distributions between groups. At the secondary functional level (Figure 8C–E), Sham and OVX groups exhibited 14 significantly divergent pathways, while Sham and OVX‐HIIT groups showed 17 differential pathways. Strikingly, OVX‐MICE mice shared near‐identical functional profiles with Sham controls, with only five pathway discrepancies. These findings suggest that MICE restores OVX‐induced GM functional perturbations, whereas HIIT lacks such regulatory capacity.

Figure 8Effect of exercise intervention on the predicted function of GM in mice with OVX‐induced osteoporosis (OP). Kyoto Encyclopedia of Genes and Genomes (KEGG) enrichment analysis was performed to predict the contributions of (A) primary and (B) secondary metabolic functions of the GM in each group of mice. Comparison of predicted functional contributions between the Sham group and (C) OVX group, (D) OVX‐HIIT group, and (E) OVX‐MICE group. *n* = 8 per group. Only the functions with significant contribution differences (*p*  < 0.05) are presented.

**Figure:**
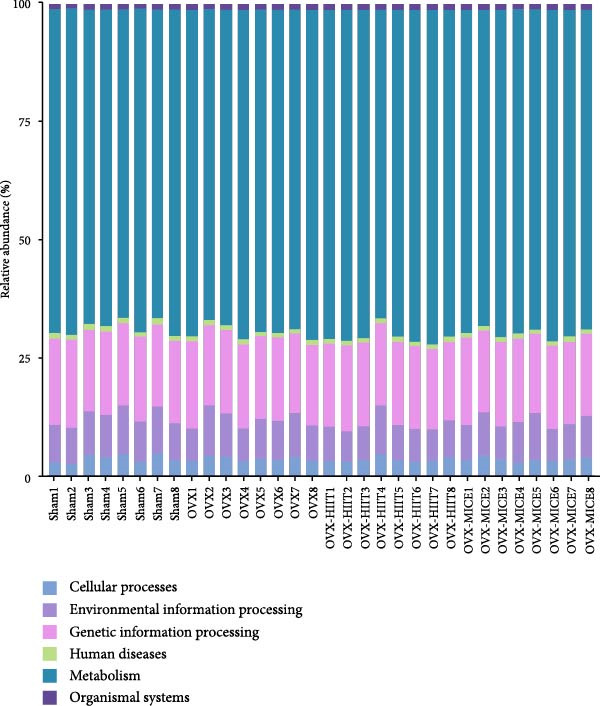
(A)

**Figure:**
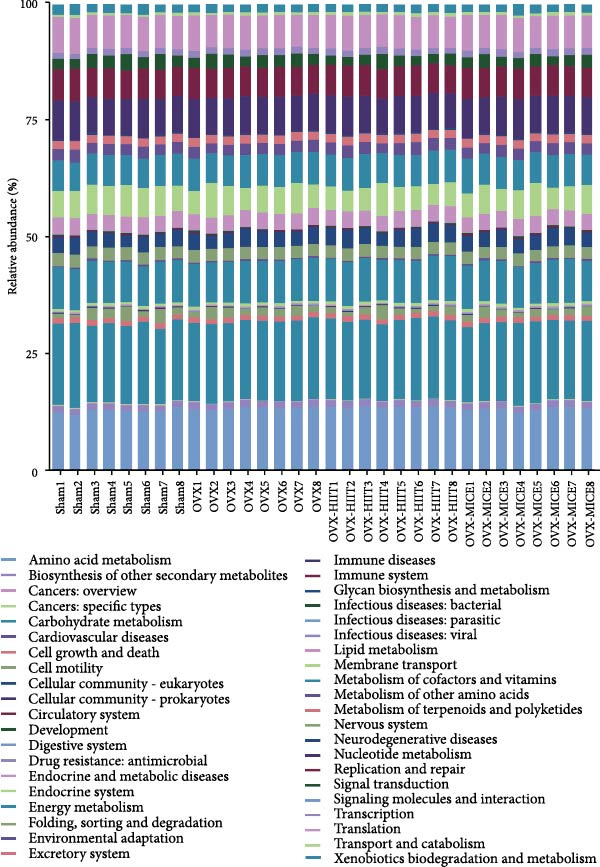
(B)

**Figure:**
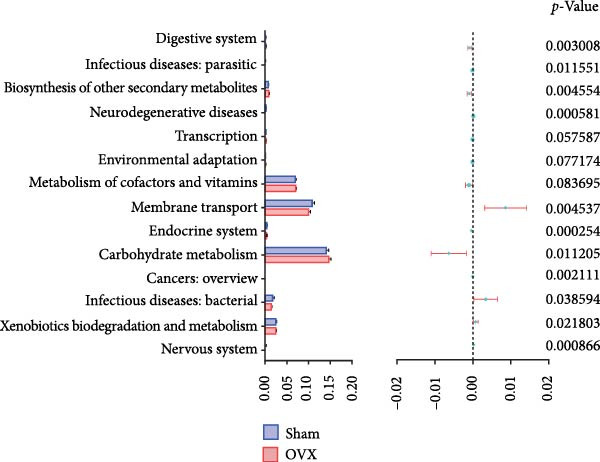
(C)

**Figure:**
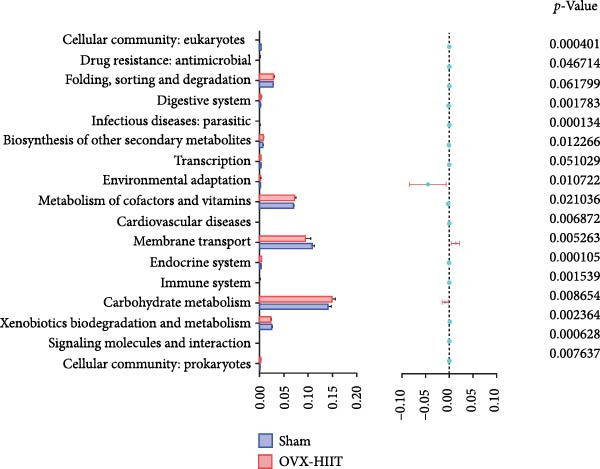
(D)

**Figure:**
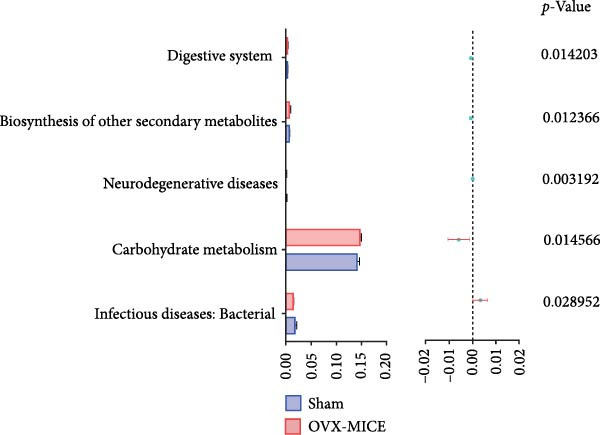
(E)

## 4. Discussion

The global rise in life expectancy and aging populations has escalated the socioeconomic burden of OP and osteoporotic fractures [44]. According to World Health Organization (WHO) estimates, individuals in developed countries face a 30%–40% lifetime risk of OP‐related fractures, imposing substantial strain on healthcare systems [45, 46]. PMOP, a primary subtype of OP, is particularly concerning due to its disproportionate impact on bone health in middle‐aged and elderly women [47].

Exercise represents a safe and effective approach to mitigate postmenopausal bone loss [48]. In this study, we compared HIIT and MICE using a resistance‐based treadmill protocol (25° incline) in OVX mice. The selection of this 25° incline was based on a twofold rationale. On the one hand, this angle is among the commonly reported treadmill inclines used in exercise studies investigating health outcomes in mice. Previous research has demonstrated that specific exercise parameters at this inclination fulfill the definitions for both HIIT and MICE protocols in rodents and that such regimens are well‐tolerated by both mice with diet‐induced obesity and OVX mice [39, 49, 50]. On the other hand, compared to low‐intensity aerobic training, resistance‐type exercise of a certain intensity has been shown to be more effective in improving BMD [51]. Therefore, this parameter was chosen to ensure a sufficient exercise stimulus within the relatively short daily session, and it was empirically observed to be well‐tolerated by the OVX mice throughout our experimental period. Our results demonstrate that MICE, but not HIIT, significantly alleviated OVX‐induced bone loss and microstructural deterioration, indicating that not all exercise modalities confer protective effects against PMOP. Mechanistically, MICE suppressed osteoclast activation by restoring GM balance, preserving intestinal barrier integrity, and inhibiting systemic release of pro‐osteoclastogenic inflammatory factors (IL‐6, IL‐1β, and TNF‐α), whereas HIIT lacked precise benefits. This finding underscores that the protective benefits against OP are modality‐specific. Regarding clinical studies in postmenopausal women, the findings are heterogeneous. Watson et al. [52, 53] conducted two randomized controlled trials demonstrating that high‐load resistance training (utilizing external weights in exercises like deadlifts, overhead presses, and back squats) was superior to home‐based, low‐intensity, low‐load resistance exercises (such as lunges, calf raises, and shrugs) in ameliorating osteosarcopenia. However, these interventions lacked consistency in exercise modality and volume between groups and were implemented without direct supervision, which are critical factors influencing the efficacy of exercise in counteracting postmenopausal bone loss [20]. Conversely, Kemmler et al. [54] suggested that the skeletal benefits of exercise in postmenopausal women might be independent of the specific exercise type. Another meta‐analysis [51] concluded that moderate‐frequency exercise was significantly more effective than high‐frequency exercise in improving BMD, yet it also cautioned that meta‐analyses can be overly blunt instruments for formulating exercise recommendations and that their results are often susceptible to publication bias. In summary, existing clinical evidence regarding optimal exercise regimens for PMOP remains contentious. Furthermore, clinical trials face inherent challenges in implementing interventions with matched exercise modalities and volumes under strictly supervised conditions, while also contending with significant inter‐individual variability. Our controlled animal study, by contrast, helps circumvent these confounding factors. Nevertheless, it is crucial to emphasize that directly extrapolating our findings from the OVX mouse model to humans has limitations and must be interpreted in conjunction with future, rigorously designed clinical studies.

Further evidence from our study indicates that the divergence in osteoprotective effects between MICE and HIIT correlated with their differential impact on systemic inflammation. While both HIIT and MICE showed a nonsignificant trend toward enhancing osteoblast activity, only MICE counteracted OVX‐induced osteoclast hyperactivity. This aligns with MICE’s superior ability to modulate the key inflammatory cytokines that drive osteoclastogenesis via the RANKL pathway. Recent studies, utilizing approaches, such as antibiotic intervention and fecal microbiota transplantation (FMT), have provided preliminary evidence that the protective effects of exercise against bone loss originate, at least partially, from its modulation of GM dysbiosis [55, 56]. Given the established role of estrogen deficiency in disrupting intestinal homeostasis and promoting systemic inflammation [57–59], our findings suggest that the differential efficacy of MICE and HIIT may stem from their distinct influences on the gut–bone axis. The GM exists throughout the intestinal lumen, and both the microbiota itself and its metabolites may serve as key antigens necessary for T lymphocyte activation under conditions of steroid hormone deficiency. Furthermore, estrogen deficiency can impair intestinal mucosal barrier function by disrupting GM homeostasis, leading to enhanced immune system reactivity [16, 60]. Our previous research also indicated that the development of PMOP is associated with GM dysbiosis and impaired intestinal barrier function. We have previously employed interventions, such as FMT, *Rothia* [37] and *Prevotella histicola* gavage [35], to modulate GM homeostasis in OVX mice, which effectively ameliorated bone microstructure damage and bone loss through the “gut–bone” axis, providing valuable evidence and insights for the regulation of this axis in the prevention and treatment of PMOP.

During mouse dissection, we observed that the intestinal walls of OVX and OVX‐HIIT groups exhibited signs of inflammation, such as swelling and a yellowish color, with some mice showing gas accumulation in the intestines along with impacted feces obstructing the distal colon. In contrast, the Sham and OVX‐MICE groups exhibited noticeably different features, with moist feces and light pink intestines. Based on these observations, we further hypothesized that the differential outcomes of HIIT and MICE in combating OVX‐induced OP may be attributed to their differing effects on osteoclast activity, inflammatory regulation, and their distinct impacts on the disruption of intestinal epithelial barrier integrity and GM homeostasis induced by OVX. Subsequent results supported our hypothesis. MICE intervention reversed the increased intestinal mucosal permeability, decreased TJPs expression, and elevated proinflammatory osteoclastogenic factor expression observed in the OVX group, whereas HIIT appeared to exacerbate these changes. These findings align with previous reports, which suggest that different types of exercise can lead to varying qualitative and quantitative changes in GM, subsequently affecting various body functions such as nutrient absorption, energy distribution, immune response, and inflammation regulation [61]. Prolonged aerobic exercise and moderate‐to‐low‐intensity resistance training can decrease the expression of inflammatory cytokines (e.g., IL‐1β and TNF‐*α*) while increasing the expression of anti‐inflammatory cytokines (e.g., IL‐4 and IL‐10) [62]. Moderate‐intensity exercise has been shown to benefit inflammatory bowel diseases, likely through exercise‐mediated modulation of GM and intestinal inflammation [63]. Meanwhile, professional athletes engaged in high‐intensity sports tend to have elevated levels of various proinflammatory factors and proteins, increased intestinal permeability, and a higher incidence of gastrointestinal diseases [63]. This may be due to the constriction of submucosal blood vessels, reduced intestinal perfusion, and enhanced intestinal permeability during intense physical exertion, leading to the ingestion of bacteria and toxins, which then triggers systemic inflammatory responses [64]. Additionally, our results indicated that MICE intervention reversed the decline in GM α‐diversity and the increase in the F/B ratio induced by OVX, whereas HIIT did not significantly affect these parameters. In the OVX‐MICE group, the enrichment of f_Prevotellaceae and s_Muribaculum_intestinale is particularly noteworthy, as both are well‐established primary degraders of dietary fiber and major producers of short‐chain fatty acids (SCFAs) [65, 66]. SCFAs, such as butyrate and acetate, are crucial for maintaining colonic health, enhancing intestinal barrier function, and exerting potent systemic anti‐inflammatory effects [67]. Meanwhile, f_Prevotellaceae was identified as the foremost differential microbiota after MICE intervention, and previous research has demonstrated that timed and quantitative administration of *Prevotella histicola* can beneficially regulate bone mass and microstructure in OVX‐induced PMOP mice [35]. Furthermore, the significant increase in g_Blautia aligns with the anti‐inflammatory phenotype observed following MICE intervention. This genus is a recognized beneficial bacterium and a key producer of acetate and other SCFAs. Its elevated abundance may have contributed to the preventive effect against estrogen deficiency‐induced OP [68]. Additionally, the expansion of the cholesterol‐metabolizing g__Eubacterium__coprostanoligenes_group suggests modulation of host cholesterol metabolism as another potential pathway through which MICE exerts its osteoprotective benefit [69].

Notably, MICE intervention significantly reduced the functional differences between the GM of OVX and Sham groups, while HIIT failed to reverse these functional disparities. These findings suggest that OVX‐induced PMOP disrupts both the composition and functionality of the GM, and MICE intervention helps restore microbial community abundance and diversity while modulating its function, whereas HIIT does not confer such beneficial effects. Prior research has also shown that compared to sedentary women, premenopausal women engaging in sustained low‐intensity exercise have an increased abundance of anti‐inflammatory bacterial species in their GM [70]. In animal studies, both voluntary wheel running and forced treadmill exercise have been shown to alter the composition and functionality of the GM, with voluntary wheel running significantly reducing the abundance of Agrobacterium in the feces, a species associated with immune dysfunction and the onset of intestinal diseases [71]. Thus, different exercise modalities have distinct impacts on GM composition, functionality, intestinal mucosal barrier integrity, and intestinal wall inflammation. These differences may, to some extent, contribute to the regulation of bone metabolism through the “gut–bone” axis, ultimately yielding varied effects on the prevention and treatment of estrogen deficiency‐induced bone loss and bone microstructural damage.

Finally, it is important to acknowledge certain limitations in this study that warrant further improvement in future research. First, this study only provides a preliminary comparison of the effects of MICE and HIIT interventions, while the effects of other exercise modalities and multimodal interventions remain to be explored. Second, our study established two distinct exercise intensity modalities in rodents. Although this exercise paradigm shares similarities with human treadmill incline training, which incorporates a partial weight‐bearing resistance component, it is crucial to note that due to differences in mass‐specific metabolic rates and inherent physiological structures between mice and humans, direct extrapolation of these findings to human exercise regimens has limitations. Our findings primarily provide mechanistic insights, and the modes, duration, and intensity of exercise interventions discussed in this study will require validation through more high‐evidence‐level clinical trials for future clinical translation. Third, the sample size of the OVX mouse model used in this study was relatively small, and larger animal trials are required for further verification. Fourth, the current limitations of 16S rRNA high‐throughput sequencing technology, such as limited resolution, lack of differentiation beyond the species level, and inability to conduct large‐scale comparative analysis, restrict its application and accuracy. Future advancements in sequencing methodologies will be necessary to optimize these analyses. Finally, it should be noted that the causal relationship within the gut–bone axis remains to be established. Although our data suggest an association between exercise‐mediated improvements in GM/barrier and bone metabolism, it is not clear whether the microbial changes directly drive the skeletal benefits. Future mechanistic studies, employing techniques, such as FMT, antibiotic depletion, or the use of germ‐free models, are necessary to confirm a direct causal link.

## 5. Conclusion

Overall, our experimental results demonstrate that MICE intervention improves bone loss and bone microstructural damage in OVX‐induced OP mice by modulating GM composition and functionality, repairing intestinal mucosal barrier damage, reducing intestinal permeability, and inhibiting the release of proinflammatory osteoclastogenic factors into the bloodstream. In contrast, HIIT does not exhibit these protective effects. Crucially, this study provides direct preclinical evidence for optimizing exercise prescriptions in PMOP. We propose that MICE should be prioritized over HIIT in the non‐pharmacological management of PMOP. Future research should focus on translating these findings into practical clinical guidelines by defining the optimal MICE parameters and validating its efficacy through high‐quality randomized controlled trials in postmenopausal women.

## Ethics Statement

The study protocol was approved by the Institutional Animal Care and Use Committee (IACUC) of the School of Medicine, Southeast University (Approval Number: 20240301034).

## Consent

The authors have nothing to report.

## Conflicts of Interest

The authors declare no conflicts of interest.

## Author Contributions

Yucheng Gao and Hao Wang completed most of the writing. Mumin Cao, Xiaoyu Liu, and Yuanwei Zhang provided assistance with the writing and searched for relevant publications. Xiangxu Chen, Yijun Rong, and Bowen Han prepared the figures. Panpan Lu, Guangchun Dai, Wenbin Fan, and Liu Shi provided input during drafting of the paper. Yingjuan Li and Yunfeng Rui conceived the idea, revised, and proofread the paper. Yucheng Gao and Hao Wang contribute equally to this work.

## Funding

This work was supported by the Winfast Charity Foundation Project (Grant YL20220525), the Jiangsu Elderly Health Research Project, the Key Project of Elderly Health Research Project (Grant LKZ2022010), and the Open Project of National Key Professional Base for Standardized Training of Resident Physicians in Zhongda Hospital Affiliated to Southeast University (Grant ZDZYJD‐QK‐2022‐7).

## Supporting Information

Additional supporting information can be found online in the Supporting Information section.

## Supporting information


**Supporting Information** The supporting information associated with this manuscript provides detailed information on the experimental protocols and reagents used in this study. Table S1 outlines the 12‐week exercise regimen, including the specific speed, duration, and total distance run for both the OVX‐MICE and OVX‐HIIT groups. Table S2 lists the primary and secondary antibodies used for IHC and western blot analysis, including the target antigens, catalog numbers, and manufacturers. Table S1. Exercise Parameters for OVX‐HIIT and OVX‐MICE Groups. Table S2. The specific information of the antibodies used.

## Data Availability

The data used during this current study are available from the corresponding author on reasonable request.
