# Supplementary material for: Gut–Bone Axis Mediates Exercise Modality‐Dependent Suppression of Inflammatory Osteoclastogenesis in Ovariectomy‐Induced Bone Loss
Source: Mediators Inflamm. 2025 Dec 12;2025:5715332. doi: 10.1155/mi/5715332 (PMC12767480; doi:10.1155/mi/5715332)
Supplement: Supplementary file 1 — Supporting Information The supporting information associated with this manuscript provides detailed information on the experimental protocols and reagents used in this study. Table S1 outlines the 12‐week exercise regimen, including the specific speed, duration, and total distance run for both the OVX‐MICE and OVX‐HIIT groups. Table S2 lists the primary and secondary antibodies used for IHC and western blot analysis, including the target antigens, catalog numbers, and manufacturers. Table S1. Exercise Parameters for OVX‐HIIT and OVX‐MICE Groups. Table S2. The specific information of the antibodies used. [file MI-2025-5715332-s001.docx]

| Exercise Regimen | | | | | |
| --- | --- | --- | --- | --- | --- |
| Week | OVX-MICE | | OVX-HIIT | | Distance(m) |
|  | Speed(m/min) | Time(min) | Speed(m/min) | Time(min) |  |
| 1 | 8 | 60 | 16 | 60 (30 rest) | 480 |
| 2 | 8 | 60 | 16 | 60 (30 rest) | 480 |
| 3 | 8 | 60 | 16 | 60 (30 rest) | 480 |
| 4 | 8 | 60 | 16 | 60 (30 rest) | 480 |
| 5 | 10 | 60 | 20 | 60 (30 rest) | 600 |
| 6 | 10 | 60 | 20 | 60 (30 rest) | 600 |
| 7 | 10 | 60 | 20 | 60 (30 rest) | 600 |
| 8 | 10 | 60 | 20 | 60 (30 rest) | 600 |
| 9 | 12 | 60 | 24 | 60 (30 rest) | 720 |
| 10 | 12 | 60 | 24 | 60 (30 rest) | 720 |
| 11 | 12 | 60 | 24 | 60 (30 rest) | 720 |
| 12 | 12 | 60 | 24 | 60 (30 rest) | 720 |

### Supplementary Material for Review and Publication

Table S1: Exercise Parameters for OVX-HIIT and OVX-MICE Groups

| **Antibodies** | **Catalog Number** | **Manufacturer** | **Country** |
| --- | --- | --- | --- |
| Osteocalcin (OCN) | 23418-1-AP | Proteintech | China |
| RUNX2 | 20700-1-AP | Proteintech | China |
| RANKL | 23408-1-AP | Proteintech | China |
| TNF-α | 60291-1-Ig | Proteintech | China |
| IL-6 | 21865-1-AP | Proteintech | China |
| IL-1β | 16806-1-AP | Proteintech | China |
| ZO-1 | ab190085 | Abcam | USA |
| Occludin | 27260-1-AP | Proteintech | China |
| Claudin-1 | 28674-1-AP | Proteintech | China |
| Horseradish per oxidase-conjugated secondary antibody | GB23303 | Servicebio | China |

Table S2: The specific information of the antibodies used.
